# Supplementary material for: An immunobiliary single-cell atlas resolves crosstalk between type 2 conventional dendritic cells and γδ T cells in cholangitis
Source: Nat Commun. 2026 Apr 10;17:3455. doi: 10.1038/s41467-026-71537-2 (PMC13076812; doi:10.1038/s41467-026-71537-2)
Supplement: Supplementary file 1 — Supplementary Information [file 41467_2026_71537_MOESM1_ESM.pdf]

Supplemental Information

An immunobiliary single-cell atlas resolves crosstalk between type 2 conventional dendritic cells and γδ T cells in cholangitis

Stefan Thomann[1], Helene Hemmer[1], Ankit Agrawal[1], Sukanya Basu[1], Judith Schaf[1], Nadine Vornberger[1], Tobias Krammer[2], Sagar[3], Fabian Imdahl[2], Tanja Poth[4], Marcell Tóth[5], Christina E. Zielinski[6,7], Tobias Poch[8,9], Jenny Krause[8,10], Andreas Rosenwald[11], Katja Breitkopf-Heinlein[12], Nuh Rahbari[13], and Dominic Grün[1,14,*]

[1]Würzburg Institute of Systems Immunology, Julius-Maximilians-Universität Würzburg, Würzburg, Germany
[2]Helmholtz Institute for RNA-based Infection Research (HIRI), Helmholtz-Center for Infection Research (HZI), Würzburg, Germany
[3]University Hospital Freiburg, Department of Gastroenterology, Hepatology, Endocrinology and Infectious Diseases, Freiburg, Germany
[4]Center for Model System and Comparative Pathology, Institute of Pathology, University Hospital Heidelberg, Heidelberg, Germany
[5]University Hospital Heidelberg, Institute of Pathology, Heidelberg, Germany
[6]Leibniz Institute for Natural Product Research and Infection Biology, Jena, Germany & Friedrich Schiller University Jena, Jena, Germany
[7]Department of Pathology, University of Cambridge, Cambridge, UK
[8]I. Department of Medicine, University Medical Center Hamburg-Eppendorf, Hamburg, Germany
[9]Present address: Cancer Immunology Program, Peter MacCallum Cancer Centre, Melbourne, VIC, Australia; Sir Peter MacCallum Department of Oncology, The University of Melbourne, Melbourne, VIC, Australia
[10]Present address: Center for Immunology, University of Minnesota, Minneapolis, Minnesota, USA
[11]Institute of Pathology, University of Würzburg, Würzburg, Germany
[12]Department of Surgery, Medical Faculty Mannheim, Heidelberg University, Mannheim, Germany
[13] Department of Surgery, University Hospital Ulm, Ulm, Germany
[14] CAIDAS - Center for Artificial Intelligence and Data Science, Würzburg, Germany
*Correspondence: dominic.gruen@uni-wuerzburg.de

Supplementary Figures 1 – 11

Supplemental Tables 1 - 9

A

sample ID	sex	age	surgical indication	scRNA-seq technology	derivation process	dissociation reagent
**MA**	m	52	liver metastasis (liposarcoma)	10x 3' v3.1 (Dual Index)	mechanical and, enzymatic dissociation, FACS	Collagenase D
**MA1**	f	52	liver metastasis (neuroendocrine tumor)	10x 3' v3.1 (Dual Index)	mechanical and, enzymatic dissociation, FACS	Collagenase D
**MA2**	f	71	in situ split timepoint colorectal liver metastasis	10x 3' v3.1 (Dual Index)	mechanical and, enzymatic dissociation, FACS	Collagenase D
**MA3**	f	57	hemi hepatectomy	10x 3' v3.1 (Dual Index)	mechanical and, enzymatic dissociation, FACS	Collagenase D
**MA4**	m	44	colorectal liver metastasis	10x 3' v3.1 (Dual Index)	mechanical and, enzymatic dissociation, FACS	Collagenase D
**MA5**	f	76	pancreatic cancer, double duct sign	10x 3' v3.1 (Dual Index)	mechanical and, enzymatic dissociation, FACS	Collagenase D

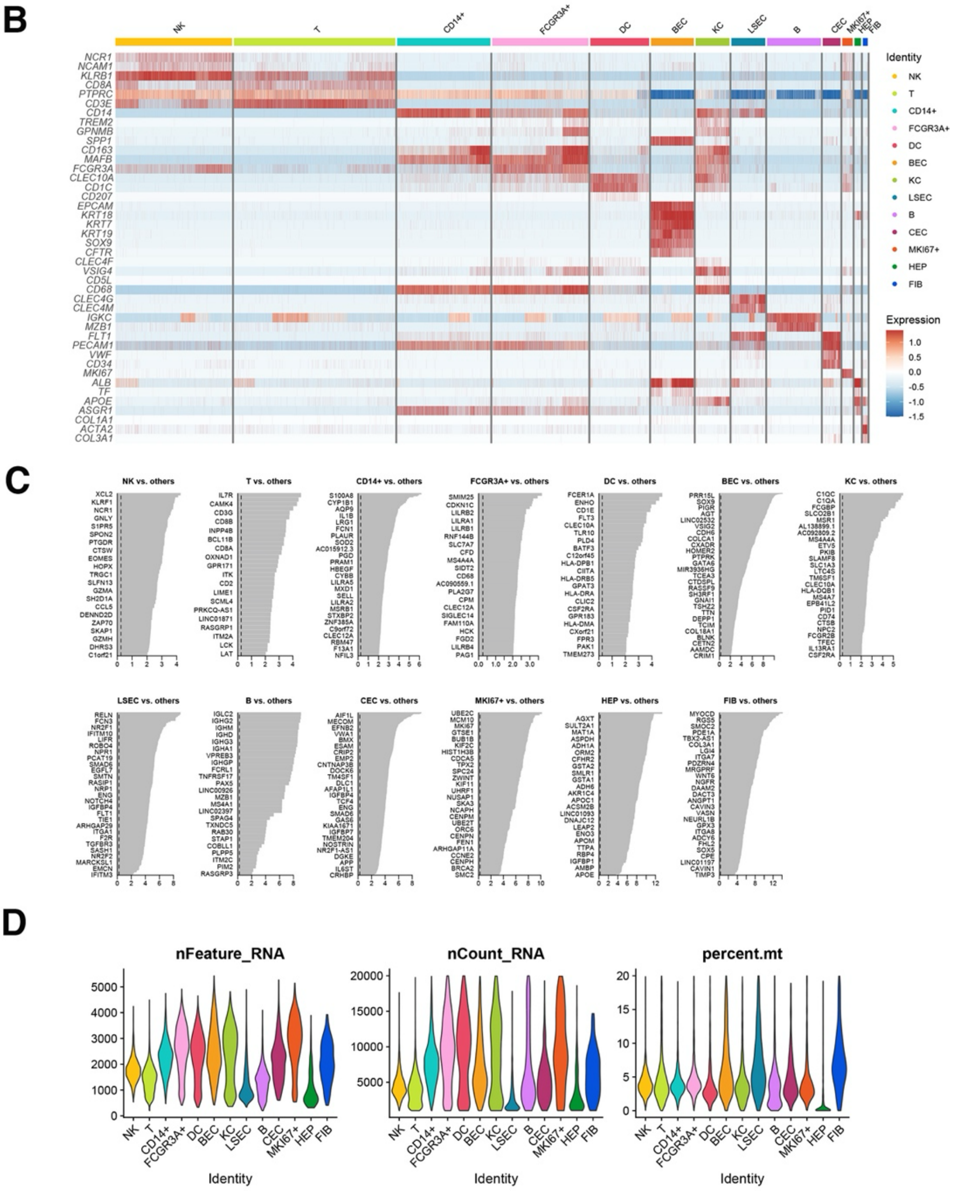

Figure S1. Characterization of human scRNA-seq data

(A) Clinical characteristics of human non-cholestatic liver disease-control samples used for scRNA-seq (compare Fig. 1B).

(B) Heatmap displaying log-normalized marker gene expression for all cell types of the human single cell data reference (n=6 patients).

(C) Barplots displaying 25 upregulated cluster-specific genes (cluster of interest vs. all other clusters, Methods).

(D) Violinplots displaying cluster-specific QC-metrics of the human scRNA-seq dataset.

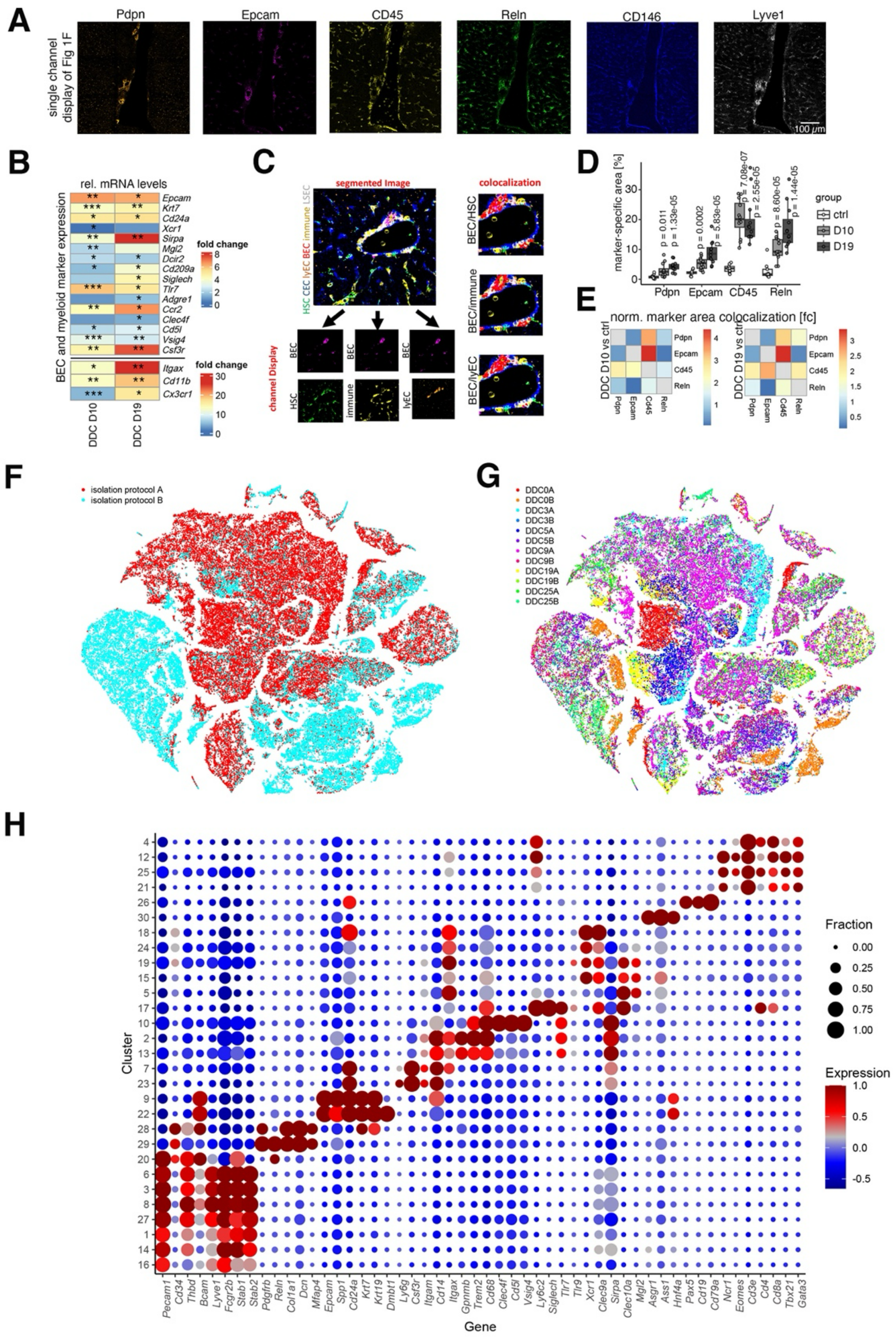
A
B
C
D
E
F
G
H
single channel display of Fig 1F
Pdpn
Epcam
CD45
Reln
CD146
Lyve1
100 µm
rel. mRNA levels
BEC and myeloid marker expression
fold change
DDC D10
DDC D19
segmented image
colocalization
channel Display
marker-specific area [%]
group
ctrl
D10
D19
norm. marker area colocalization [fc]
DDC D10 vs ctrl
DDC D19 vs ctrl
isolation protocol A
isolation protocol B
Fraction
Expression
Cluster
Gene

Figure S2. Immunobiliary niche in DDC-treated mice and DDC scRNA-seq data

(A) Channel-specific IF display of the immunobiliary niche on steady state mouse liver tissue (compare main Fig. 1F).

(B) Heatmap displaying relative mRNA levels (qPCR) of cell type marker genes in DDC D10/D19 liver tissues normalized to liver control. Two sided t-test (n=4 mice/group). Asterisks indicate level of significance.

(C) Graph displaying IF-inferred channel-specific co-localization from intensity-based segmentation in the portal niche. Region of interest (right) highlights colocalized marker areas in red.

(D) Boxplot displaying marker-specific area quantification of segmented IF images derived from control (ctrl), DDC D10 and D19 liver tissues (Fig. 1F,I). Two-sided t-test (ctrl, DDC D10: n=3 mice/group, DDC D19: n=4 mice; 3 images per mouse).

(E) Heatmap displaying average colocalization foldchange (fc) within different color channels in DDC D10, D19 liver tissues normalized to control.

(F) tSNE representation of the cell composition of the DDC atlas data based on isolation protocol A/B (Methods).

(G) tSNE representation of the independent samples of the DDC atlas data. A/B indicates the isolation strategy of the respective timepoint (Methods).

(H) Dotplot displaying candidate genes for the DDC single cell atlas reference for clusters with at least 200 cells. Color code encodes for Z score of the mean expression across clusters and dot size represents fractions of cells being positive.

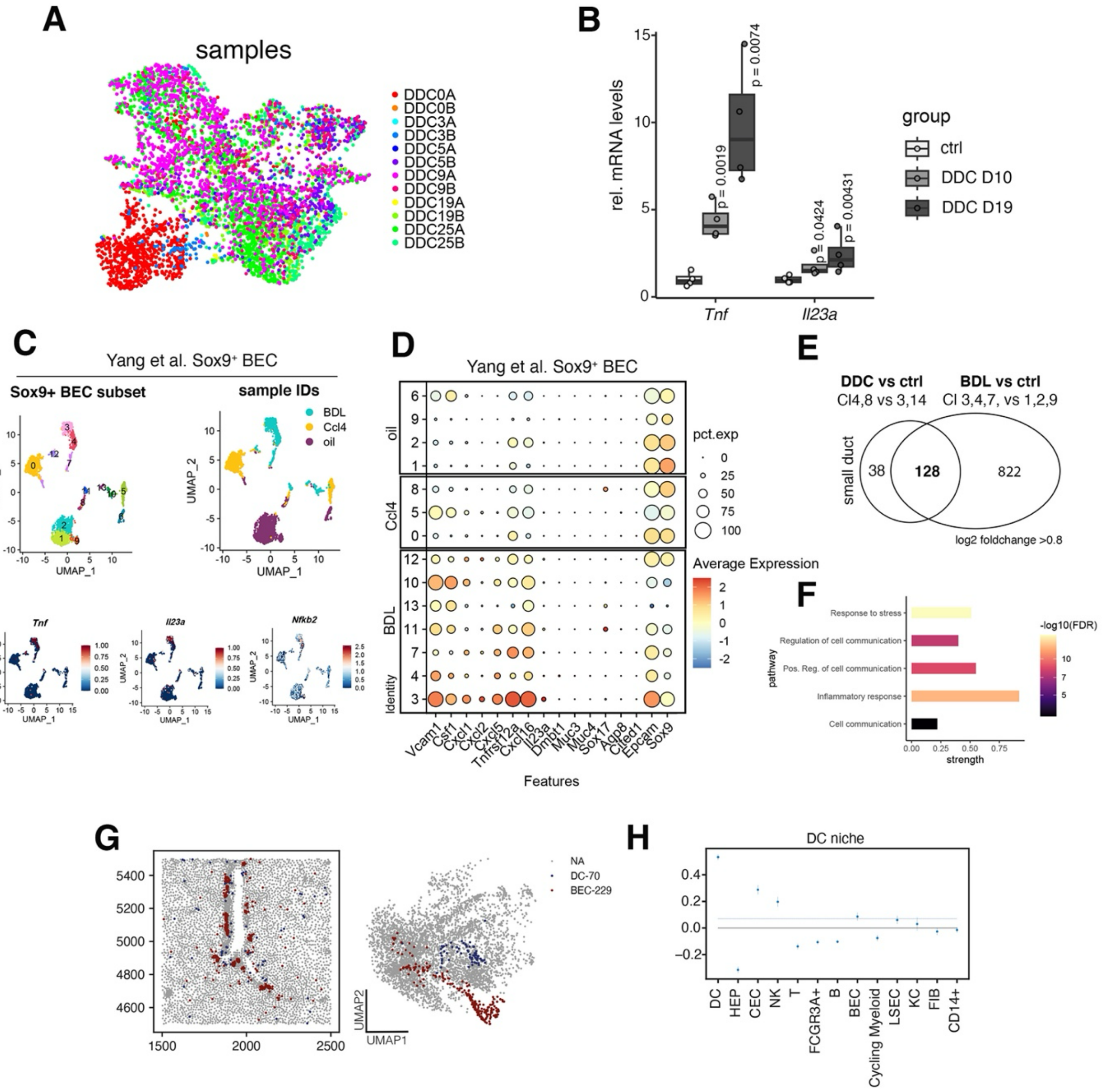

Figure S3. DDC induces ductular reaction and small duct inflammatory disease

(A) UMAP representation of sample-specific labeling of the BEC subset.

(B) Boxplot displaying relative *Tnf* and *Il23a* mRNA levels (qPCR) in DDC D10/D19 liver tissues normalized to liver control. One sided t-test (n=4 mice/group).

(C) UMAP of Sox9⁺ BEC clusters (left) and treatment conditions (right) of BDL and CCl4 treated mice. "Oil" represents respective control. UMAP of log-normalized expression of *Tnf*, *Il23a*, *Nfkb2*. Data derived from (Yang et al., 2021).

(D) Dotplot displaying BEC cluster-specific gene expression of the BDL Sox9⁺ data subset derived from (Yang et al., 2021). Log-normalized average expression and fraction of cells expressing gene of interest shown. BDL derived cluster 3 was identified as Sox9+ BEC cluster with pro-inflammatory changes.

(E) Venn diagram displaying commonly upregulated genes (log2-foldchange > 0.8) in Sox9⁺ BDL and DDC data.

(F) Pathway enrichment analysis of the common BDL/DDC-upregulated genes.

(G) Region of interest (same as in Figure 1A) displaying colocalized DC and BEC niche.

(H) Logistic regression coefficients of the DC niche. Regression coeffcients (y-axis) were ordered by magnitude. Grey dotted line indicates threshold (c=0.07) for plotting the cell type niche interaction map in Figure 2F. Error bars indicate SD from five cross fold trainings of logistic regression.

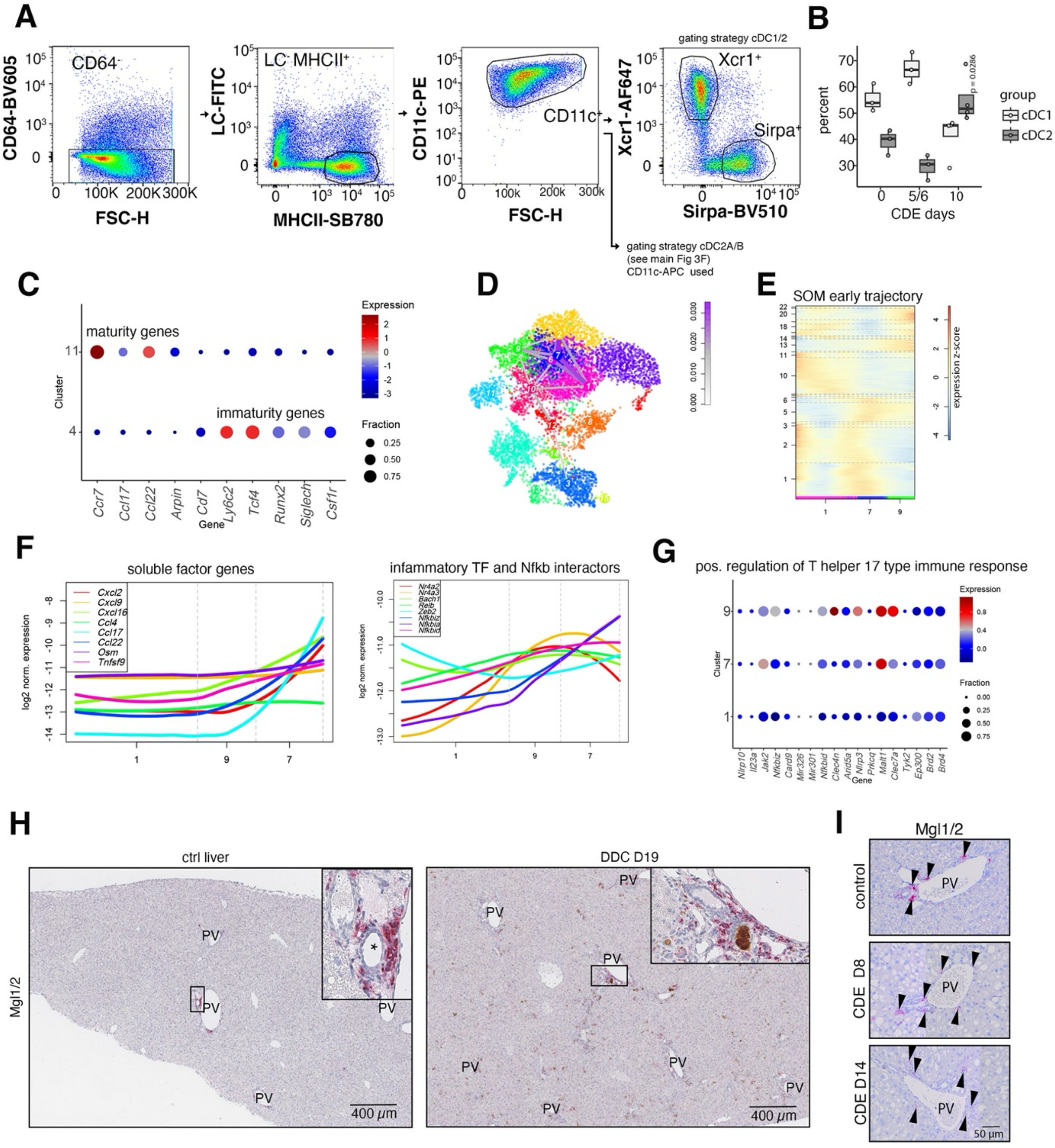
A
CD64-BV605
CD64⁻
FSC-H
LC-FITC
LC⁻ MHCII⁺
MHCII-SB780
CD11c-PE
CD11c⁺
FSC-H
gating strategy cDC1/2
Xcr1-AF647
Xcr1⁺
Sirpa⁺
Sirpa-BV510
gating strategy cDC2A/B
(see main Fig 3F)
CD11c-APC used
B
percent
group
cDC1
cDC2
p = 0.0286
CDE days
C
maturity genes
immaturity genes
Cluster
Expression
Fraction
Gene
Ccr7
Ccl17
Ccl22
Arpin
Cd7
Ly6c2
Tcf4
Runx2
Siglech
Csf1r
D
E
SOM early trajectory
expression z-score
F
soluble factor genes
log2 norm. expression
Cxcl2
Cxcl9
Cxcl16
Ccl4
Ccl17
Ccl22
Osm
Tnfsf9
infammatory TF and Nfkb interactors
Nr4a2
Nr4a3
Bach1
Relb
Zeb2
Nfkbiz
Nfkbia
Nfkbid
G
pos. regulation of T helper 17 type immune response
Expression
Fraction
Cluster
Gene
H
ctrl liver
DDC D19
Mgl1/2
PV
400 µm
I
Mgl1/2
control
CDE D8
CDE D14
PV
50 µm

Figure S4. Disease stage-specific cell state transition of cDC2 reflects immature niche restoration

(A) FACS gating strategy used to quantify cDC1 and cDC2 derived from [1] (compare Fig. 3B). Arrow indicates alternative gating for cDC2A/B quantification (compare Fig. 3F).

(B) Boxplot displaying cDC1/cDC2 FACS-based quantification at D0, CDE D5/6 and D10. One-sided Wilcoxon test (CDE D5/6: n=3mice per group, CDE D10: n=4 mice; two independent experiments). Control datapoints ("0") derive from Fig. 3B.

(C) Dotplot displaying cDC2-specific gene expression of maturation associated genes. Log-transformed gene expression is color coded and fraction of cells expressing the gene is encoded by dot size.

(D) UMAP of cDC2 subset overlaid with VarID2-derived transition probabilities.

(E) Pseudotime-ordered self-organizing map (SOM) of the inferred early disease trajectory (cluster 1 – 7 – 9).

(F) Pseudotemporal gene expression profiles of representative soluble factor (left) or proinflammatory and Nfkb interacting genes (right). The sum of gene expression across all cells was normalized to one.

(G) Dotplot displaying cDC2 cluster-specific gene expression with the annotation "positive regulation of T helper 17 type immune response". Z score of the mean expression is color coded and fraction of cells expressing the gene is encoded by dot size.

(H) Low resolution overview of Mgl1/2 immunohistochemical staining in control (ctrl) and DDC D19 liver. Box indicates region of interest with higher resolution. Chromogenic signal is highly specific to portal fields containing portal veins (PV) and bile ducts (asterisk). Scale bar, 400 µm.

(I) Immunohistochemical staining of Mgl1/2 in control, CDE D8, CDE D14 liver tissues. Arrowheads highlight Mgl1/2$^+$ cells. Scale bar, 50 µm.

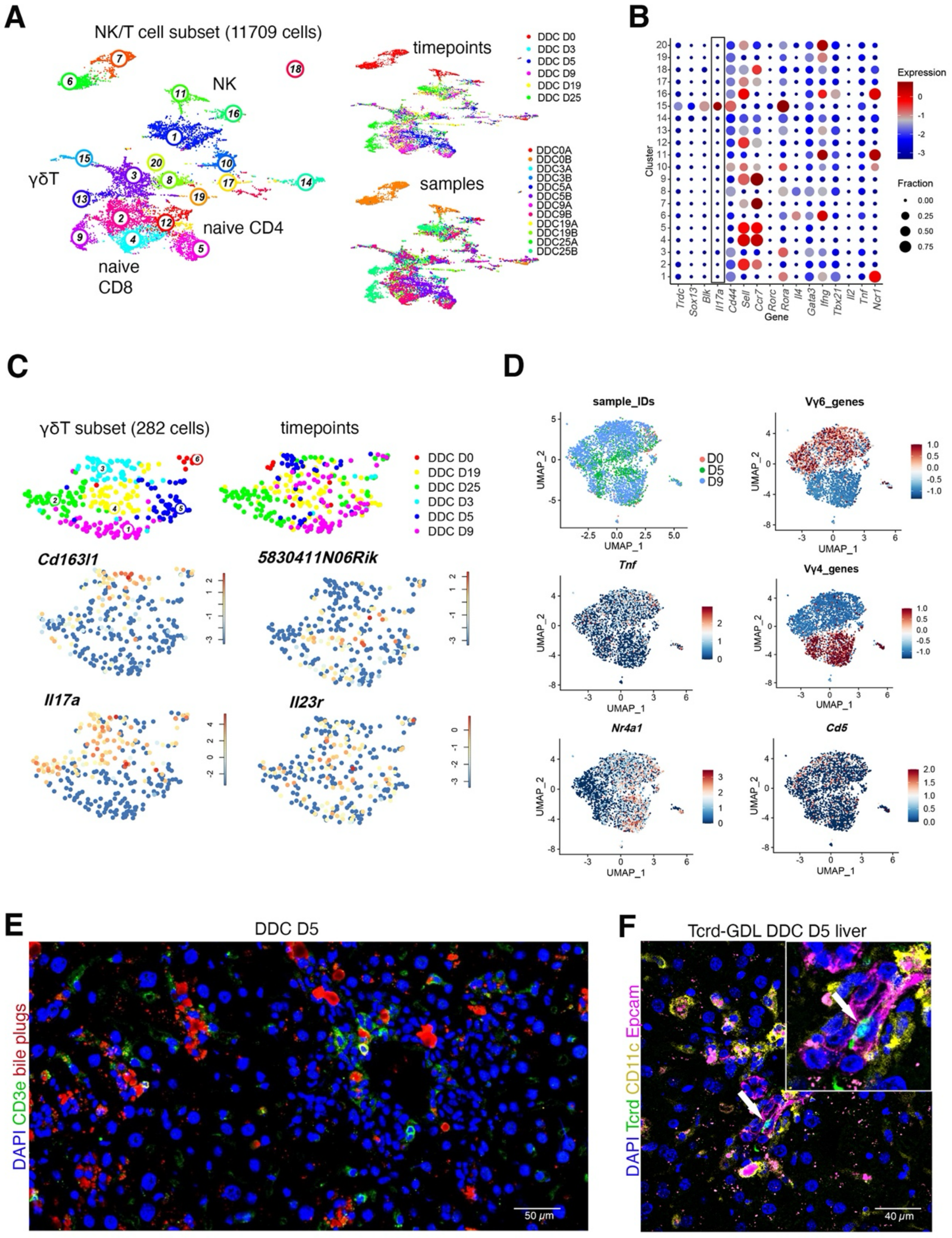

A
NK/T cell subset (11709 cells)
NK
γδT
naive CD4
naive CD8
timepoints
DDC D0
DDC D3
DDC D5
DDC D9
DDC D19
DDC D25
samples
DDC0A
DDC0B
DDC3A
DDC3B
DDC5A
DDC5B
DDC9A
DDC9B
DDC19A
DDC19B
DDC25A
DDC25B
B
Cluster
Gene
Trdc Sox13 Blk Il17a Cd44 Sell Ccr7 Rorc Rora Il4 Gata3 Ifng Tbx21 Il2 Tnf Ncr1
Expression
Fraction
C
γδT subset (282 cells)
timepoints
DDC D0
DDC D19
DDC D25
DDC D3
DDC D5
DDC D9
Cd163l1
5830411N06Rik
Il17a
Il23r
D
sample_IDs
D0
D5
D9
Vγ6_genes
Tnf
Vγ4_genes
Nr4a1
Cd5
UMAP_1
UMAP_2
E
DDC D5
DAPI CD3e bile plugs
50 μm
F
Tcrd-GDL DDC D5 liver
DAPI Tcrd CD11c Epcam
40 μm

Figure S5. *Scart1*⁺ and *Scart2*⁺ γδ T cells produce profibrogenic Il17a in a niche-dependent manner

(A) UMAP of DDC T/NK cell subset (11,709 cells) with cluster, timepoint and sample annotation.

(B) Dotplot displaying NK/T cell cluster-specific gene expression. Log-normalized gene expression is color-coded and fraction of cells expressing the gene is encoded by dot size. Box highlights *Il17a*, which is exclusively expressed in cluster 15.

(C) UMAP representation of γδ T cell subset (282 cells) with cluster annotation and individual timepoints (top). Log-normalized expression of *Scart1* (Cd163l1), *Scart2* (5830411N06Rik), *Il17a* and *Il23r*.

(D) UMAP representation displaying timepoint-specific data distribution in the γδ T17 subset (top left). Log-normalized expression of Vγ6/Vγ4 genes and *Tnf, Nr4a1, Cd5* in γδ T17 cells.

(E) IF displaying DAPI and CD3e co-stained DDC D5 liver derived from C57BL/6J mice. Scale bar, 50 µm.

(F) IF displaying DAPI, CD11c and Epcam co-stained DDC D5 liver derived from Tcrd-GDL mice. Arrow indicates peribiliary localized EGFP$^+$ cell. Scale bar, 40 µm.

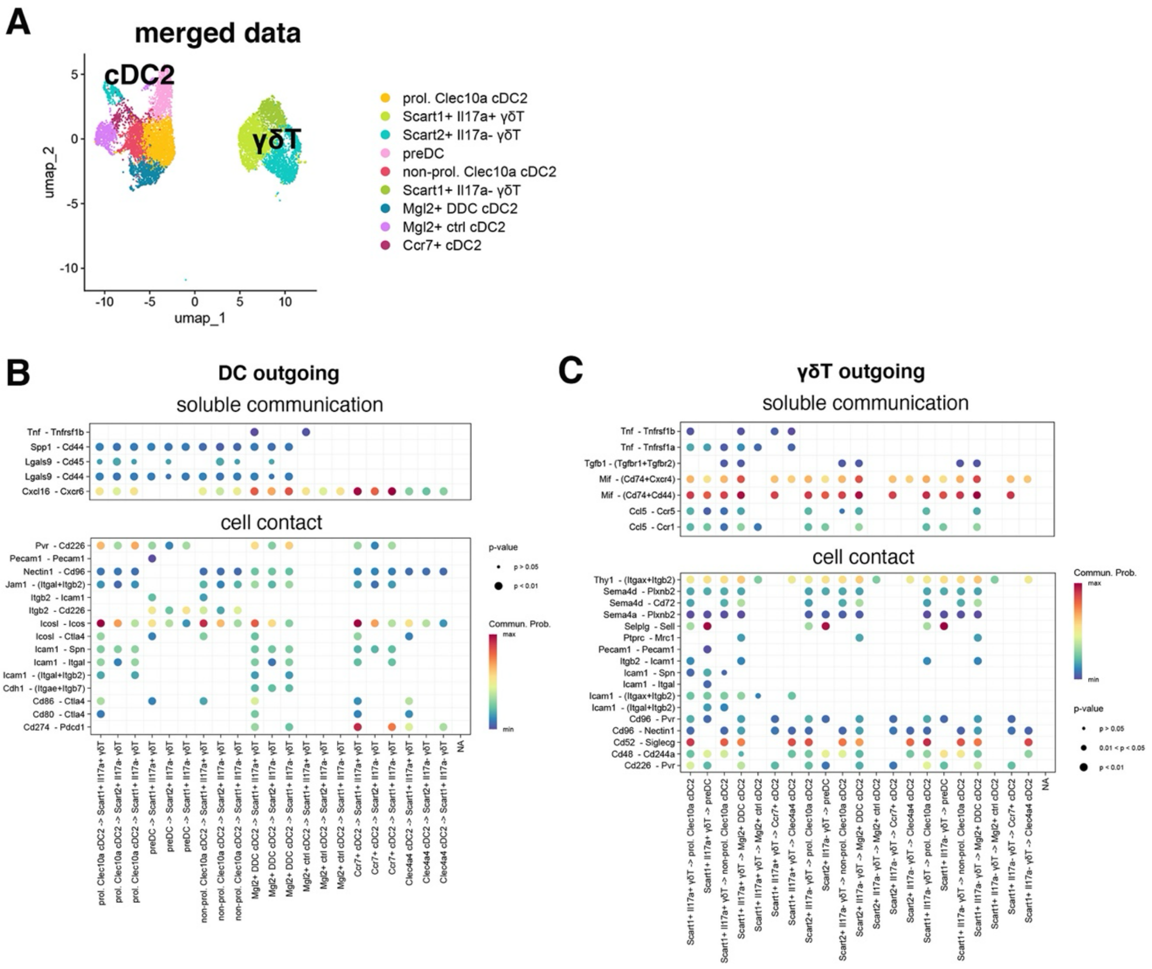

Figure S6. Ligand-receptor interaction analysis of cDC2-γδ T cells

(A) UMAP representation of merged liver γδ T cell and cDC2 displaying clusters used for cell communication inference.

(B) Dotplot displaying cDC2 outgoing molecular cell contact and soluble communication pairs. Communication probability encoded as color code and p-values as dot size. Statistics derived from permutation test.

(C) Dotplot displaying g γδ T outgoing molecular cell contact and soluble communication pairs. Communication probability encoded as color code and p-values as dot size. Statistics derived from permutation test.

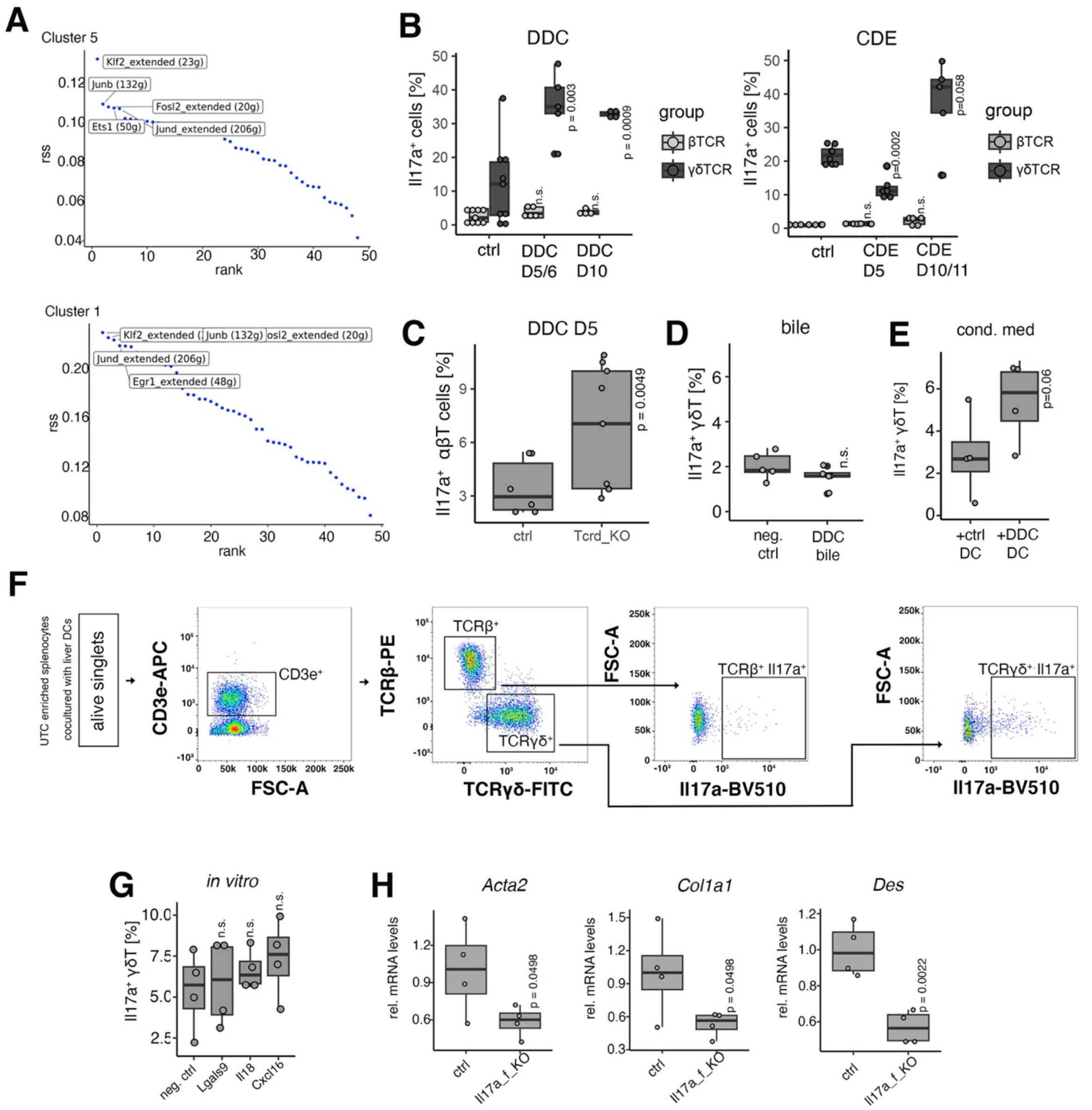
A
Cluster 5
Klf2_extended (23g)
Junb (132g)
Fosl2_extended (20g)
Ets1 (50g)
Jund_extended (206g)
rss
rank
Cluster 1
Jund_extended (206g)
Egr1_extended (48g)
B
DDC
Il17a+ cells [%]
ctrl
DDC D5/6
DDC D10
p = 0.003
p = 0.0009
n.s.
group
βTCR
γδTCR
CDE
CDE D5
CDE D10/11
p=0.0002
p=0.058
C
DDC D5
Il17a+ αβT cells [%]
ctrl
Tcrd_KO
p = 0.0049
D
bile
Il17a+ γδT [%]
neg. ctrl
DDC bile
E
cond. med
+ctrl DC
+DDC DC
p=0.06
F
UTC enriched splenocytes cocultured with liver DCs
alive singlets
CD3e-APC
FSC-A
CD3e+
TCRβ-PE
TCRγδ-FITC
TCRβ+
TCRγδ+
TCRβ+ Il17a+
Il17a-BV510
TCRγδ+ Il17a+
G
in vitro
neg. ctrl
Lgals9
Il18
Cxcl16
H
Acta2
Col1a1
Des
rel. mRNA levels
ctrl
Il17a_f_KO
p = 0.0498
p = 0.0022

Figure S7. $\gamma\delta$ T cell niche-dependent Il17a production

(A) Rank visualization of *Scart2⁺* $\gamma\delta$ T17 cluster-specific regulon specificity scores (rss). Top 5 regulons are indicated.

(B) Boxplots displaying frequency of Il17a$^+$ cells within Cd3$^+$ β TCR$^+$ (light grey) and Cd3$^+$ $\gamma\delta$ TCR$^+$ (dark grey) compartments in DDC (left) and CDE (right) diet models after cytokine re-stimulation. Two-sided t-test (4 (DDC), 5 (CDE) independent experiments; DDC: ctrl (n=9 mice), D5/6 (n=5 mice), D10 (n=4 mice); CDE: ctrl (n=8 mice), D5 (n=6 mice), D10 (n=5 mice).

(C) Boxplots displaying proportion of Il17a$^+$ cells within Cd3e$^+$ β TCR$^+$ cells after cytokine re-stimulation in control (ctrl) and Tcrd_KO mice. Two-sided Wilcoxon test (three independent experiments, ctrl: n=6 mice, Tcrd_KO: n=9 mice).

(D) Boxplots displaying frequency of Il17a$^+$ cells within Cd3$^+$ $\gamma\delta$ TCR$^+$ cells after in vitro administration of DDC D5 derived bile. One-sided t-test (n=5, two independent experiments).

(E) Boxplots displaying frequency of Il17a$^+$ cells within Cd3$^+$ $\gamma\delta$ TCR$^+$ cells after in vitro administration of ctrl and DDC D5 DC-derived conditioned medium. One-sided t-test (4 independent experiments).

(F) FACS gating strategy of UTC enriched cocultures to quantify Il17a$^+$ β TCR$^+$ and Il17a$^+$ $\gamma\delta$ TCR$^+$ cells.

(G) Boxplots displaying frequency of Il17a$^+$ cells within Cd3$^+$ $\gamma\delta$ TCR$^+$ cells after in vitro administration of Lgals9, Il18, Cxcl16. One-sided t-test (4 independent experiments).

(H) Boxplots displaying relative *Acta2, Col1a1* and *Des* mRNA levels (qPCR) in DDC D16 treated ctrl and Il17a_f_KO liver tissues normalized to liver control. One-sided t-test (n=4 mice/group).

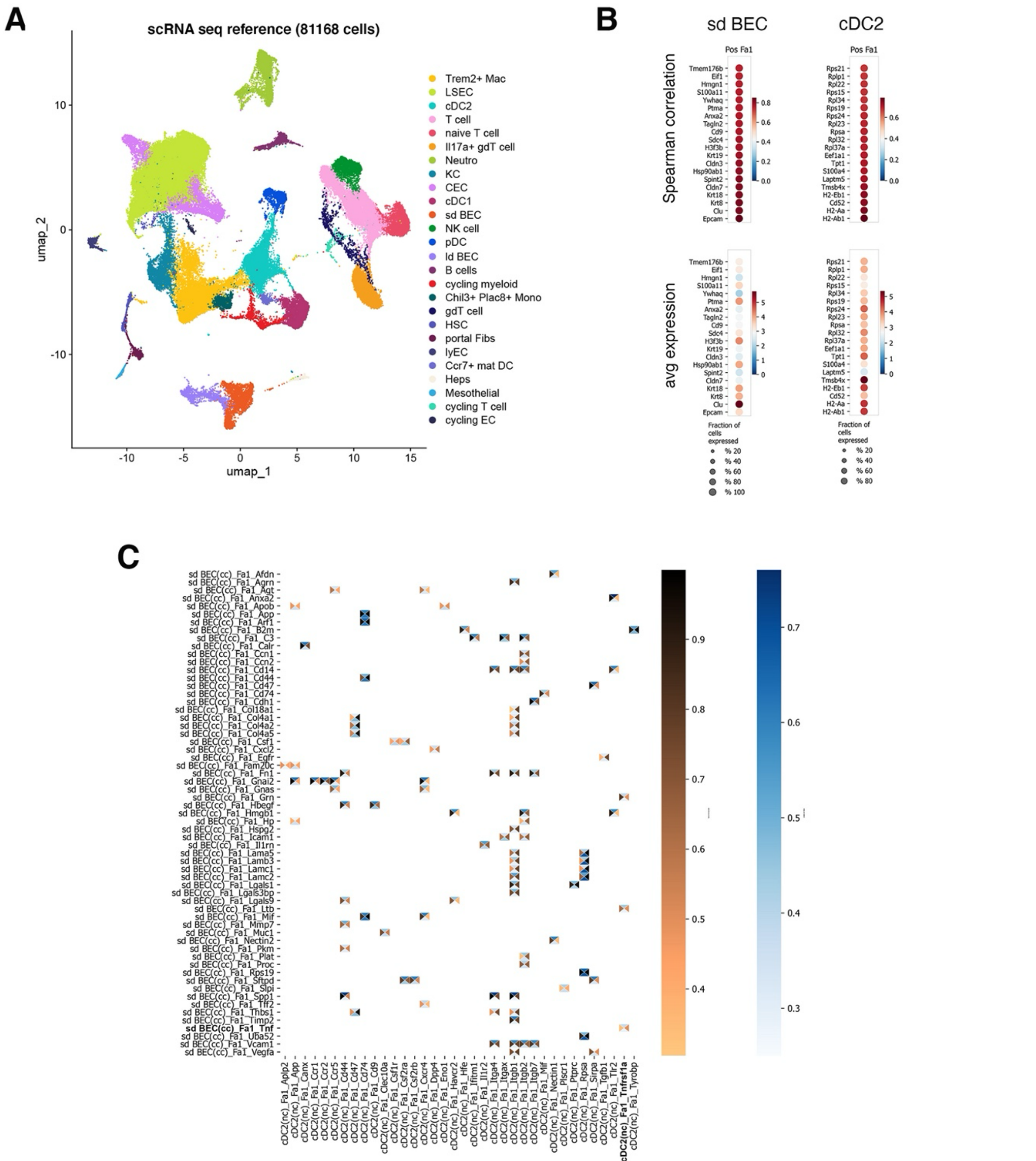

Figure S8. Spatial niche deconvolution resolves cDC2B and γδ T cell containing cholangitis-associated tissue domains

(A) UMAP representation of merged DDC atlas (Fig. 1K) and liver γδ T cell data (Fig. 4A) containing cell annotation used for NiCo label transfer.

(B) Dotplot displaying Spearman correlation analysis (top) and average expression (bottom) of the top 20 positively correlated genes from sd BEC and cDC2 (Fa 1).

(C) Ligand-receptor pairs correlated with covarying BEC Fa1 and cDC2 Fa2 (cc, central cell; nc, niche cell). The rectangle’s north and south faces represent ligand and receptor correlation of the factors, while west and east faces represent the proportion of cells expressing the respective ligands or receptors. Gene correlation threshold of 0.25 and gene expression threshold of 0.3 was applied. Ligand, y-axis, receptors x-axis.

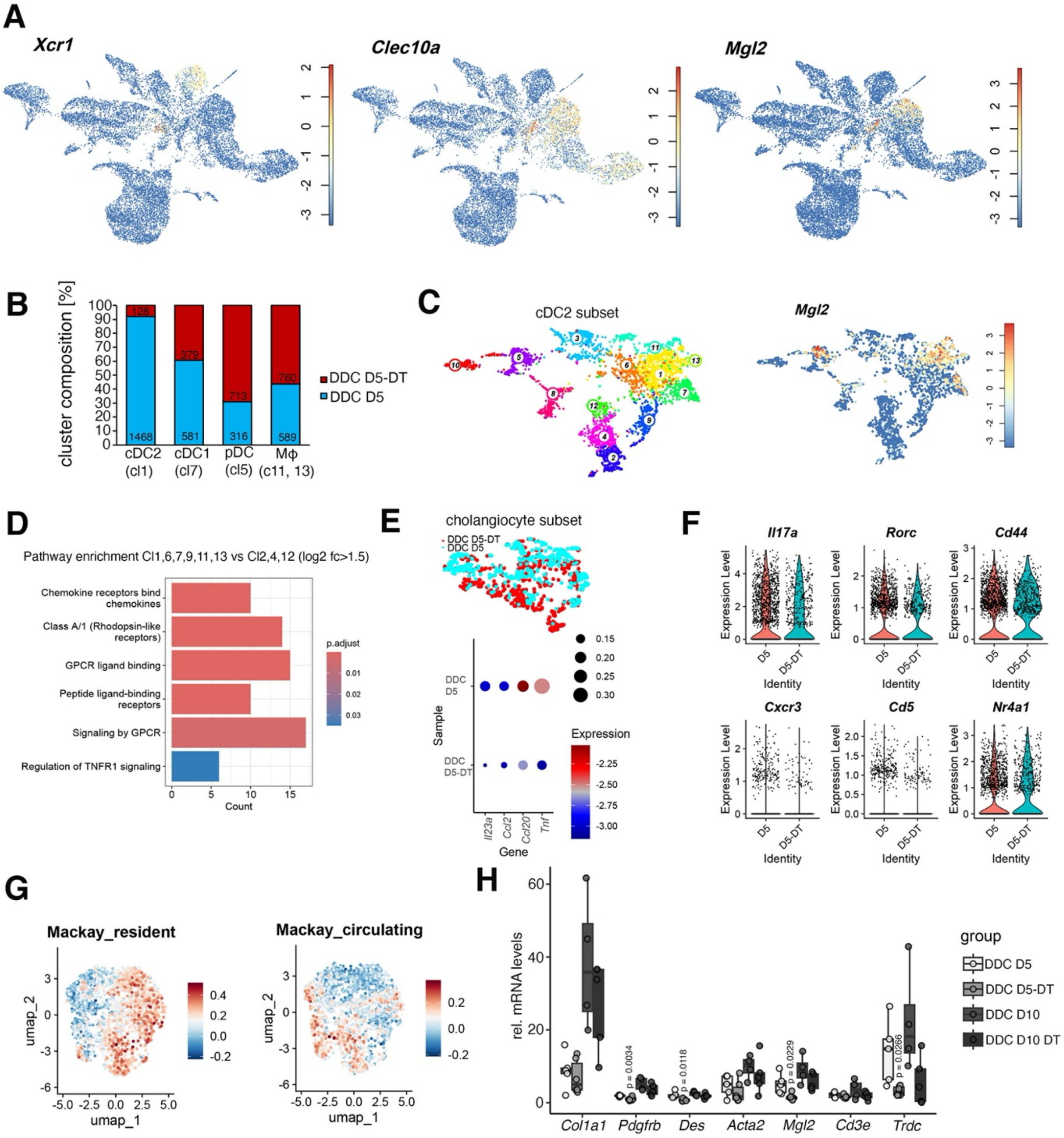

A
Xcr1
Clec10a
Mgl2
B
cluster composition [%]
cDC2 (cl1)
cDC1 (cl7)
pDC (cl5)
Mφ (c11, 13)
DDC D5-DT
DDC D5
C
cDC2 subset
Mgl2
D
Pathway enrichment Cl1,6,7,9,11,13 vs Cl2,4,12 (log2 fc>1.5)
Chemokine receptors bind chemokines
Class A/1 (Rhodopsin-like receptors)
GPCR ligand binding
Peptide ligand-binding receptors
Signaling by GPCR
Regulation of TNFR1 signaling
Count
p.adjust
E
cholangiocyte subset
DDC D5-DT
DDC D5
Sample
Gene
Il23a
Ccl2
Ccl20
Tnf
Expression
F
Il17a
Rorc
Cd44
Cxcr3
Cd5
Nr4a1
Expression Level
Identity
D5
D5-DT
G
Mackay_resident
Mackay_circulating
umap_1
umap_2
H
rel. mRNA levels
Col1a1
Pdgfrb
Des
Acta2
Mgl2
Cd3e
Trdc
p = 0.0034
p = 0.0118
p = 0.0229
p = 0.0266
group
DDC D5
DDC D5-DT
DDC D10
DDC D10 DT

Figure S9. Early cDC2B depletion results in reduced $\gamma\delta$ T17 effector differentiation

(A) UMAP representation of the combined DDC D5/DDC D5-DT dataset displaying log-normalized gene expression of *Xcr1, Clec10a, Mgl2*.

(B) Stacked barplot displaying relative contribution of DDC D5 (blue) and DDC D5-DT (red) samples to cell type-specific clusters in the merged DDC D5/DDC D5-DT data.

(C) UMAP representation displaying clusters of the cDC2 subset of merged DDC D5 and DDC D5-DT data (left). UMAP representation of cDC2 subset displaying log-normalized *Mgl2* expression (right).

(D) Barplot displaying pathway enrichment of differentially upregulated genes in cDC2 in DDC D5 (clusters 1,3,6,7,9,11,13) vs. DDC D5-DT (clusters 2,4,12). Enriched pathways in the non-depleted condition show an association with cDC2 function.

(E) UMAP representation of BEC subset of merged DDC D5 and DDC D5-DT data (left). Dotplot displaying expression of cholestasis associated inflammatory mediators. Log-normalized expression is color coded and fraction of cells expressing the gene is encoded by dot size (right).

(F) Violinplots displaying log-normalized expression if *Il17a*, *Rorc*, *Cd44*, *Cxcr3*, *Cd5*, *Nr4a1* within $\gamma\delta$ T17 subset across samples (compare Fig. 6F).

(G) UMAP representation displaying log-normalized gene expression of Mackay resident and circulatory gene signatures in $\gamma\delta$ T17 subset derived from experiment of Fig. 6F.

(H) Boxplot displaying tissue-derived transcript levels in DDC D5, DDC D5-DT, DDC D10, DDC D10-DT conditions. Data normalized to control liver tissue transcript levels. One sided t test between D5 and D5-DT conditions (ctrl: n=4 mice, DDC D5: n=5 mice, DDC D5-DT: n=6mice, DDC D10: n=4 mice, DDC D10-DT: n=5 mice).

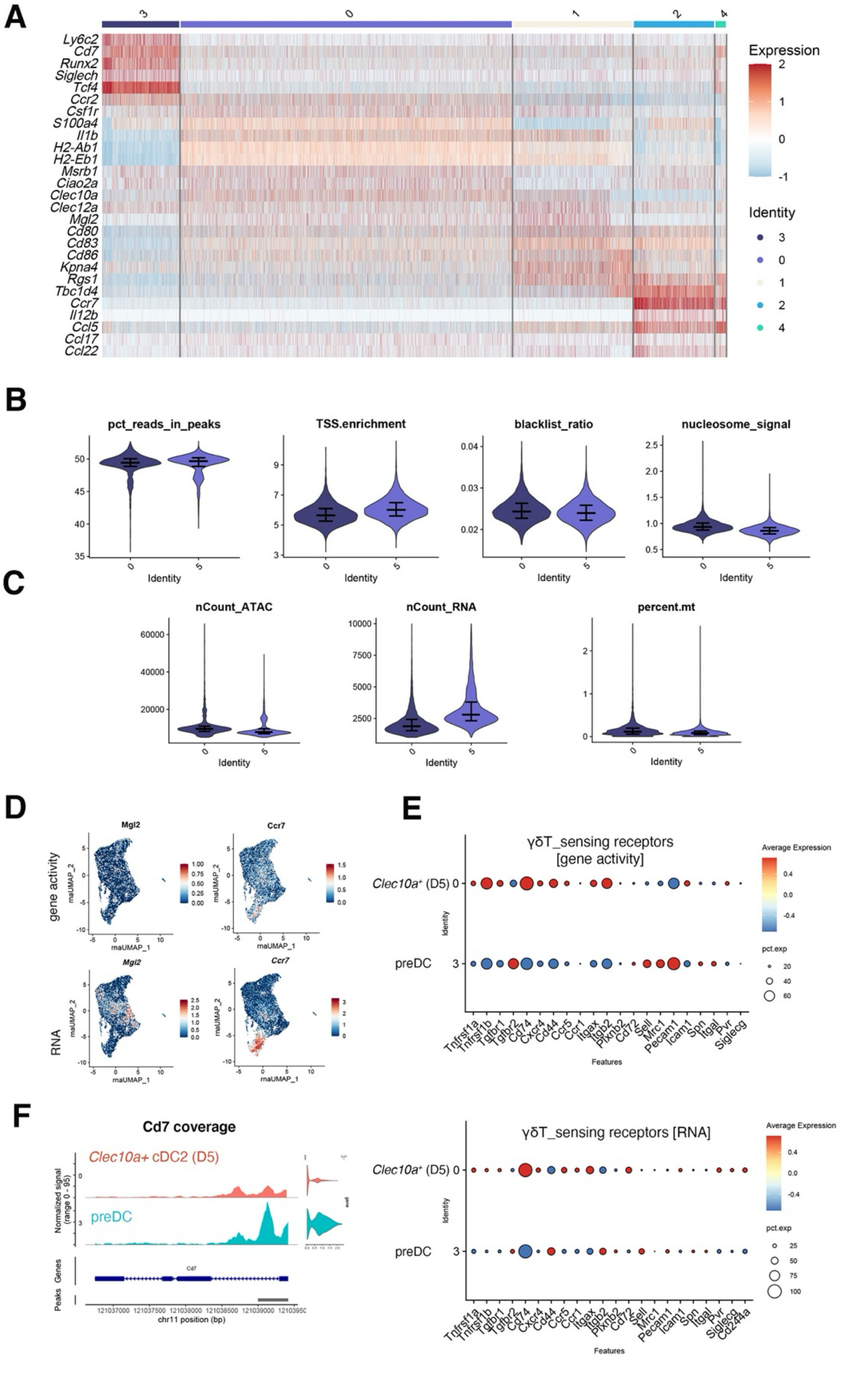

A
Ly6c2
Cd7
Runx2
Siglech
Tcf4
Ccr2
Csf1r
S100a4
Il1b
H2-Ab1
H2-Eb1
Msrb1
Ciao2a
Clec10a
Clec12a
Mgl2
Cd80
Cd83
Cd86
Kpna4
Rgs1
Tbc1d4
Ccr7
Il12b
Ccl5
Ccl17
Ccl22
Expression
Identity
B
pct_reads_in_peaks
TSS.enrichment
blacklist_ratio
nucleosome_signal
Identity
C
nCount_ATAC
nCount_RNA
percent.mt
Identity
D
Mgl2
Ccr7
gene activity
RNA
rnaUMAP_1
rnaUMAP_2
E
γδT_sensing receptors [gene activity]
Clec10a⁺ (D5) 0
preDC 3
Average Expression
pct.exp
Identity
Features
Tnfrsf1a
Tnfrsf1b
Tgfbr1
Tgfbr2
Cd74
Cxcr4
Cd44
Ccr5
Ccr1
Itgax
Itgb2
Plxnb2
Cd72
Sell
Mrc1
Pecam1
Icam1
Spn
Itgal
Pvr
Siglecg
F
Cd7 coverage
Clec10a+ cDC2 (D5)
preDC
Normalized signal (range 0 - 95)
Genes
Peaks
chr11 position (bp)
γδT_sensing receptors [RNA]
Clec10a⁺ (D5) 0
preDC 3
Average Expression
pct.exp
Features
Cd244a

Figure S10. Quality control metrics of Multiome data

(A) Heatmap displaying log-normalized marker gene expression of the cDC2 subset data (compare with Figure 6I).

(B) Violin plots displaying QC metrics of the ATAC-seq data derived from the DDC D0/D5 Multiome cDC2 data subset. Bars within violinplots display median and interquartile range.

(C) Violin plots displaying captured ATAC/RNA counts and fraction of reads mapped to the mitochondrial genome of the Multiome cDC2 data subset. Bars within violinplots display median and interquartile range.

(D) scRNA-derived UMAP (rnaUMAP) highlighting gene activity levels inferred from scATAC data (top) and *Mgl2* and *Ccr7* RNA expression (bottom).

(E) Dotplot displaying $\gamma\delta$ T cell sensing receptor gene activities in cDC2 across clusters 3 (preDC) and 0 (Clec10a^{+} cells). Log-normalized mean expression is color coded and fraction of cells expressing the gene is encoded by dot size.

(F) Cd7 coverage plot displaying gene-specific pseudobulk accessibility tracks in preDC (cluster 3) and Clec10a^{+} cells (cluster 0). Genomic region of interest and coordinates displayed in the bottom row. Violinplot on the right displays *Cd7* RNA expression.

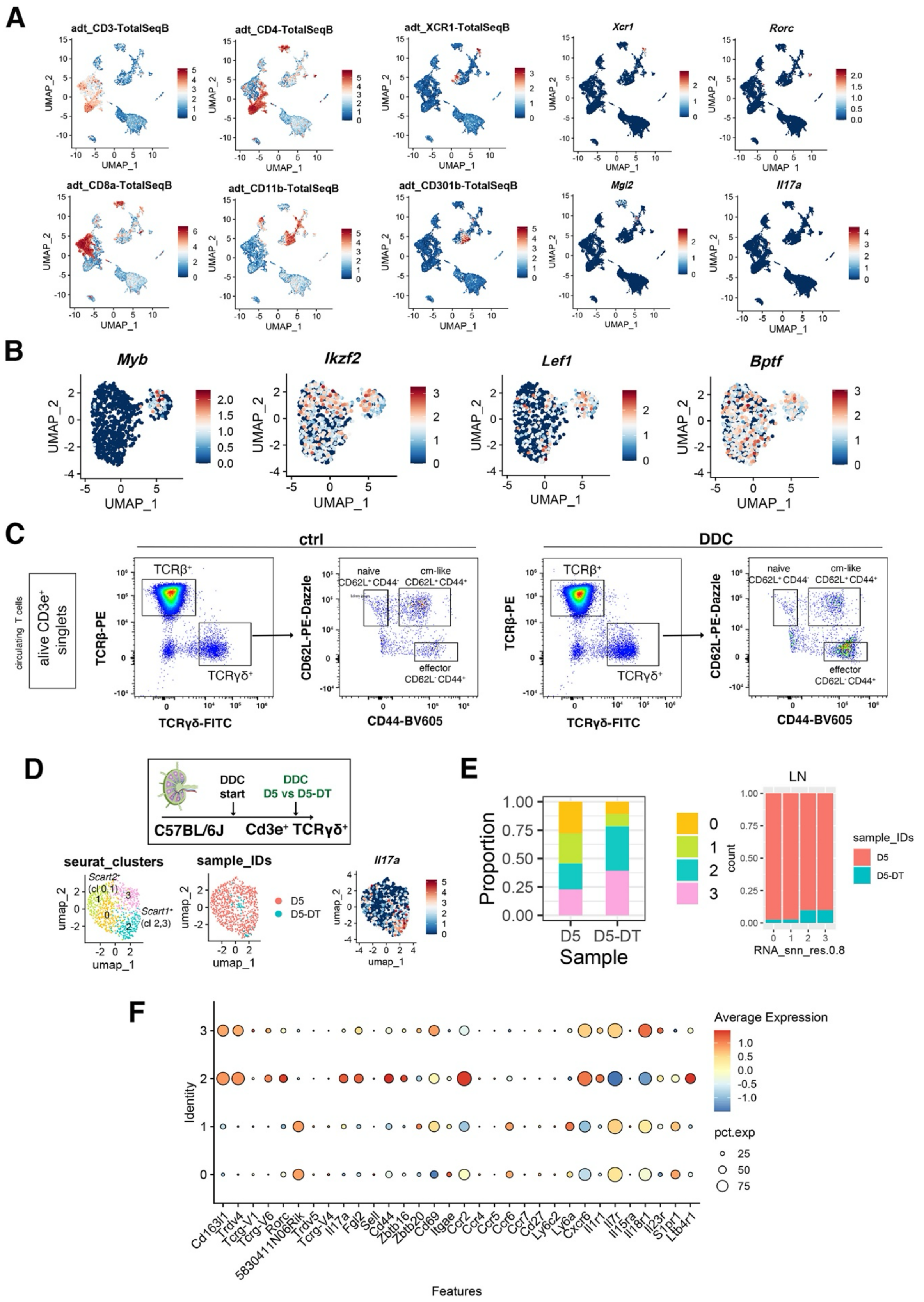
A
adt_CD3-TotalSeqB
adt_CD4-TotalSeqB
adt_XCR1-TotalSeqB
Xcr1
Rorc
adt_CD8a-TotalSeqB
adt_CD11b-TotalSeqB
adt_CD301b-TotalSeqB
Mgl2
Il17a
UMAP_1
UMAP_2
B
Myb
Ikzf2
Lef1
Bptf
C
ctrl
DDC
circulating T cells
alive CD3e+ singlets
TCRβ+
TCRγδ+
TCRβ-PE
TCRγδ-FITC
naive CD62L+ CD44-
cm-like CD62L+ CD44+
effector CD62L- CD44+
CD62L-PE-Dazzle
CD44-BV605
D
DDC start
DDC D5 vs D5-DT
C57BL/6J
Cd3e+ TCRγδ+
seurat_clusters
Scart2+ (cl 0,1)
Scart1+ (cl 2,3)
sample_IDs
D5
D5-DT
Il17a
umap_1
umap_2
E
Proportion
Sample
LN
count
sample_IDs
RNA_snn_res.0.8
F
Average Expression
pct.exp
Identity
Features
Cd163l1
Trdv4
Tcrg-V1
Tcrg-V6
Rorc
5830411N06Rik
Trdv5
Tcrg-V4
Il17a
Fgl2
Sell
Cd44
Zbtb16
Zbtb20
Cd69
Itgae
Ccr2
Ccr4
Ccr5
Ccr6
Ccr7
Cd27
Ly6c2
Ly6a
Cxcr6
Il1r1
Il7r
Il15ra
Il18r1
Il23r
S1pr1
Ltb4r1

Figure S11. γδ T cells in liver draining lymph nodes

(A) UMAP of the complete LN dataset displaying centered log-ratio normalized protein data (CD3, CD4, CD8, CD11b, XCR1, CD301b) and log-normalized RNA data (*Xcr1*, *Mgl2*, *Rorc*, *Il17a*).

(B) UMAP representation displaying log-normalized *Myb, Lef1, Ikzf2, Bptf* expression.

(C) FACS gating strategy exemplified for an untreated control and DDC sample shown. Circulating γδ T cells were subtyped into naïve ($CD62^{+}$ $CD44^{-}$), central memory (cm)-like ($CD62L^{+}$ $CD44^{+}$) and effector ($CD62L^{-}$ $CD44^{+}$) subsets.

(D) Experimental design and structure of the merged liver draining LN DDC D5 and LN DDC D5-DT dataset (top). UMAP representations of clusters, samples and log-normalized Il17a expression (bottom).

(E) Stacked barplot displaying relative proportions of γδ T17 per cluster (left) and condition (right).

(F) Dotplot displaying cluster-specific gene expression in γδ T cells. Log-normalized average expression and fraction of cells expressing gene of interest shown.

Gene	Ensembl ID	Probesets
**Ptprc**	ENSMUSG00000026395	8
**Cxcr4**	ENSMUSG00000045382	8
**Ms4a1**	ENSMUSG00000024673	8
**Irf4**	ENSMUSG00000021356	8
**Ccr2**	ENSMUSG00000049103	8
**Cd14**	ENSMUSG00000051439	8
**Cd68**	ENSMUSG00000018774	8
**Csf1r**	ENSMUSG00000024621	8
**Cx3cr1**	ENSMUSG00000052336	8
**Cxcl14**	ENSMUSG00000021508	8
**Itgam**	ENSMUSG00000030786	8
**Gpm6a**	ENSMUSG00000031517	8
**Mki67**	ENSMUSG00000031004	8
**Cxcl2**	ENSMUSG00000058427	8
**Cxcl5**	ENSMUSG00000029371	8
**Krt7**	ENSMUSG00000023039	8
**Vcam1**	ENSMUSG00000027962	8
**Yap1**	ENSMUSG00000053110	8
**Ccr7**	ENSMUSG00000037944	8
**Cd209a**	ENSMUSG00000031494	8
**Cd83**	ENSMUSG00000015396	8
**Clec9a**	ENSMUSG00000046080	8
**Clec10a**	ENSMUSG00000000318	8
**Itgae**	ENSMUSG00000005947	8
**Itgax**	ENSMUSG00000030789	8
**Ly6c2**	ENSMUSG00000022584	8
**Mgl2**	ENSMUSG00000040950	8
**Siglech**	ENSMUSG00000051504	8
**Tlr7**	ENSMUSG00000044583	8
**Xcr1**	ENSMUSG00000060509	8
**5830411N06Rik**	ENSMUSG00000054672	8
**Cd163l1**	ENSMUSG00000025461	8
**Cd44**	ENSMUSG00000005087	8
**S1pr1**	ENSMUSG00000045092	8
**Sell**	ENSMUSG00000026581	8
**Trdv4**	ENSMUSG00000076867	7
**Ccn1**	ENSMUSG00000028195	8
**Ccn2**	ENSMUSG00000019997	8
**Myc**	ENSMUSG00000022346	8
**Tead1**	ENSMUSG00000055320	8
**Tead4**	ENSMUSG00000030353	8
**Tgfbr2**	ENSMUSG00000032440	8
**Adh4**	ENSMUSG00000037797	8
**Afp**	ENSMUSG00000054932	8
**Asgr1**	ENSMUSG00000020884	8
**Bche**	ENSMUSG00000027792	8
**Ces3b**	ENSMUSG00000062181	8
**Cyp2a4**	ENSMUSG00000074254	8
**Cyp2f2**	ENSMUSG00000052974	8
**Glul**	ENSMUSG00000026473	8
**Gpc3**	ENSMUSG00000055653	8
**Hal**	ENSMUSG00000020017	8
**Saa1**	ENSMUSG00000074115	6
**Acta2**	ENSMUSG00000035783	8
**Col1a1**	ENSMUSG00000001506	8
**Ednra**	ENSMUSG00000031616	8
**Lox**	ENSMUSG00000024529	8
**Pdgfra**	ENSMUSG00000029231	8
**Cd200r2**	ENSMUSG00000090176	8
**Cd3g**	ENSMUSG00000002033	8
**Cd69**	ENSMUSG00000030156	8
**Cxcr6**	ENSMUSG00000048521	8
**Gzma**	ENSMUSG00000023132	8
**Il2ra**	ENSMUSG00000026770	8
**Il7r**	ENSMUSG00000003882	8
**Tbx21**	ENSMUSG00000001444	8
**Zfp683**	ENSMUSG00000049410	8
**Il23r**	ENSMUSG00000049093	8
**Cd163**	ENSMUSG00000008845	8
**Kcna2**	ENSMUSG00000040724	8
**Timd4**	ENSMUSG00000055546	8
**Vsig4**	ENSMUSG00000044206	8
**Vsig8**	ENSMUSG00000049598	8
**Fcgr3**	ENSMUSG00000059498	8
**Gpnmb**	ENSMUSG00000029816	8
**Dmbt1**	ENSMUSG00000047517	8
**Ckap2l**	ENSMUSG00000048327	8
**Clec4g**	ENSMUSG00000074491	8
**Cxcl10**	ENSMUSG00000034855	8
**Esm1**	ENSMUSG00000042379	8
**Kng1**	ENSMUSG00000022875	8
**Ccl21a**	ENSMUSG00000095320	4
**Pdpn**	ENSMUSG00000028583	8
**Bmp4**	ENSMUSG00000021835	8
**Bmx**	ENSMUSG00000031377	8
**Edn1**	ENSMUSG00000021367	8
**Vegfc**	ENSMUSG00000031520	8
**Il6ra**	ENSMUSG00000027947	8
**Trem1**	ENSMUSG00000042265	8
**Cd27**	ENSMUSG00000030336	8
**Eomes**	ENSMUSG00000032446	8
**Ncr1**	ENSMUSG00000062524	8
**Klrb1c**	ENSMUSG00000030325	8
**Reln**	ENSMUSG00000042453	8
**Svep1**	ENSMUSG00000028369	8
**Cd3e**	ENSMUSG00000032093	8
**Cd4**	ENSMUSG00000023274	8
**Pdcd1**	ENSMUSG00000026285	8
**Il18r1**	ENSMUSG00000026070	8
**Rorc**	ENSMUSG00000028150	8

Supplemental Table 1. Add-on gene list and designed probes per gene for spatial transcriptomics detection.

Experimental models: mouse strains		
C57BL/6J	C57BL/6JRj	Janvier<br>RRID:IMSR_RJ:C57BL-6JRJ
CD301b-DTR (Mgl2-DTR) [3]	B6(FVB)Mgl2tm1.1(HBEGF/EGFP)Aiwsk/J	#023822 (Jackson),<br>RRID:IMSR_JAX:023822
Il17a_f_KO [4]	B6.129P2-Il17atm1Yiw Il17ftm2Yiw	#RRID: MGI:3830065
Tcrdtm_KO [5]	B6.129P2-Tcrd tm1 Mom/J	#002120 (Jackson),<br>RRID:IMSR_JAX:002120
Tcrd- GDL [6]	C57BL/6N-Trdctm1(EGFP/HBEGF/luc)Impr/J	Strain #:038040<br>RRID:IMSR_JAX:038040

Supplemental Table 2. Mouse strains used in this study.

Strain	Allele	Primer	Sequence (5'-3')
**CD301b-DTR**	Mgl2	17980	TGATTGTCCCAGAAGCCTTG
		17981	CTCTCTGAGGCCCTTGAATG
**Tcrd- GDL**	TCRd	oIMR7623	GGTTCCAGCAGCTAGCCCTCT
		13007	ACACCGGCCTTATTCCAAG
		65209	AACATGGAAAGCTGTACTCAACTC
		65210	TGAGTGTCCTTCCTTTCGGTA
**Tcrd-KO**	TCRd	oIMR6916	CTTGGGTGGAGAGGCTATTC
		oIMR6917	AGGTGAGATGACAGGAGATC
		oIMR8744	CAAATGTTGCTTGTCTGGTG
		oIMR8745	GTCAGTCGAGTGCACAGTTT
**Il17a/f_KO**	Il17a	IL17_P214	CTTTCAGGGTCGAGAAGATGCTG
		IL17_P215	AAGCAGTTTGGGACCCCTTTACA
		IL17_P216	ATCCAATCCCCCATCACCTT
		Il17_P217	GTTGGGACTTGCCATTCTGA

Supplemental Table 3. Primer sequences used for genotyping.

Critical commercial assays		
**REAGENT/RESOURCE**	**Source/Company**	**Identifier**
AccuStart Gel Track PCR Super Mix	Quantabio	P/N 84228
Arcturus PicoPure RNA Isolation Kit	appliedbiosystems	REF 12204-01
CD3e MicroBead Kit mouse	MiltenyiBiotec	130-094-973
CD4/CD8 (TIL) MicroBeads mouse	MiltenyiBiotec	130-116-480
Epcam MicroBeads	MiltenyiBiotec	130-105-958
F4/80 MicroBeads	MiltenyiBiotec	130-110-443
CD11c MicroBeads	MiltenyiBiotec	130-125-835
CD146 MicroBeads	MiltenyiBiotec	130-092-007
LS Columns	MiltenyiBiotec	130-042-401
LD Columns	MiltenyiBiotec	130-042-901
Qubit 1x dsDNA HS Assay Kit	ThermoFisher	Q33231
Revert Aid H Minus First Strand cDNA synthesis kit	ThermoFisher	K1632
Superfrost Plus Adhesion Microscope Slides	MENZEL-Gläser	J1800AMNZ
iTaq Universal SYBR Green Supermix	BioRad	1725121
RNAscope 2.5 HD Duplex Detection Kit (Chromogenic)	Bio-Techne	322500
Chromium Next GEM Single Cell 3’ GEM, Library and Gel Bead Kit v3.1	10x Genomics	PN-1000121
Chromium Next GEM Single Cell 3’ kit v3.1	10x Genomics	PN-1000268
3’ Feature Barcode Kit	10x Genomics	PN-1000262
Chromium Next GEM Chip G Single Cell Kit	10x Genomics	PN-1000120
Single Index Kit T Set A	10x Genomics	PN-1000213
Dual Index Kit TT Set A	10x Genomics	PN-1000215
Dual Index Kit NT Set A	10x Genomics	PN-1000242
Xenium Mouse Multi-Tissue Atlassing Panel	10x Genomics	PN-1000627
Xenium Decoding Consumables	10x Genomics	PN-1000487
Xenium Decoding Reagents	10x Genomics	PN-1000461
Xenium Slides & Sample Prep reagents	10x Genomics	PN-1000460
Xenium Cell Segmentation Add-On Kit	10x Genomics	PN-1000662

Supplemental Table 4. Critical commercial assays.

Chemicals, peptides, and recombinant proteins		
**REAGENT/RESOURCE**	**Source/Company**	**Identifier**
ACK lysis buffer	Thermo Scientific	A1049201
Brefeldin A	Merck (Sigma)	#7651
BSA	Fisher Scientific	BP9703-100
choline-deficient chow	Ssniff	-
DDC-enriched chow (0.1%)	Ssniff	-
Collagenase	Sigma	C2674
Collagenase D	Roche	11088858001
mouse recombinant Cxcl16	Peprotech	250-28
5% Digitonin	Invitrogen	BN2006
Diphtheriatoxin (unnicked)	EMD Millipore	322326
DL-Ethionine	Sigma	E5139
DMSO	Serva	39757.02
EB buffer	Qiagen	19086
Ethanol absolute	Serva	200-578-6
Fluoromount-DAPI	SouthernBiotech	0100-20
FCS	Corning	35-016-CV
FOXP3/Transcription Factor Staining Buffer Set	eBioscience	REF 00-5523-00
Glycerol Solution	Ricca	3290-16
Histodenz	Sigma	D2158
Ionomycin	Merck (Sigma)	#10634
Methanol	Sigma	34860
mouse recombinant Lgals9	R&D systems	3535-GA-050
mouse recombinant IL18	Biolegend	767004
mouse recombinant IL23	Thermo Scientific	14-8231-63
mouse recombinant TNF-a	Biolegend	575202
Pen Strep	Gibco	15140-122
PMA	Merck (Sigma)	P8139
Pronase	Roche	10165921001
RNase Inhibitor Murine	New England Biolabs	M0314S
RPMI1640	Gibco	21875-034
10% Tween-20	BioRad	1662404
Zombie-NIR Fixable Viability Kit	Biolegend	423106

Supplemental Table 5. Chemicals, peptides, and recombinant proteins.

REAGENT/ RESOURCE	Source/Company	Identifier	Used for
**Anti-mouse antibodies (clone)**			
CD3e (145-2C11)	BD Biosciences	#553061, RRID:AB_394594	FACS/IF
CD11b (M1/70)	Affymetrix eBioscience	#11-0112-82, RRID:AB_464935	FACS/IF
CD11c (N418)	BioLegend	#117309, RRID:AB_313778	FACS/IF
CD16/32	Biolegend	#101330, RRID:AB_2561482	FACS
CD19(MB19-1)	BioLegend	#101506, RRID:AB_312825	FACS
CD31 (MEC13.3)	BioLegend	#102429, RRID:AB_2566206	FACS/IF
CD44 (IM7)	BioLegend	#103047, RRID:AB_2562451	FACS
CD45 (30-F11)	BioLegend	#103108, RRID:AB_312972	FACS/IF
CD62L (MEL-14)	BioLegend	#104447, RRID:AB_2566162	FACS
CD64 (X54-5/7.1)	BioLegend	#139323, RRID:AB_2629778	FACS
CD146 (ME-9F1)	BD Biosciences	#740095, RRID:AB_2739854	FACS/IF
CD172a/SIRPα (P84)	BioLegend	#144023, RRID:AB_2650815	FACS/IF
CD278 (ICOS)	eBioscience	# 14-9949-82, RRID:AB_468637	functional
CD301b (URA-1)	Biolegend	#146803, RRID:AB_2562943	FACS/IF
CD335 (NKp46)	BioLegend	#137605, RRID:AB_2149150	FACS
EPCAM (G8.8)	eBioscience	#17-5791-82, RRID:AB_2716944	FACS/IF
F4/80 (REA126)	MiltenyiBiotec	#130-117-509, RRID:AB_2727970	FACS/IF
Il17a (TC11-18H10.1)	BioLegend	#506933, RRID:AB_2562668	FACS
LYVE-1 (ALY7)	eBioscience	#53-0443-82, RRID:AB_1633415	FACS/IF
MGL1/2	R&D systems	#AF4297, RRID:AB_2248147	IHC
MHC class 2 (M5/114.15.2)	eBioscience	#78-5321-82, RRID:AB_2744917	FACS
PDGFRB	Cell Signaling	#3169, RRID:AB_2162497	IHC
PDPN (eBio8.1.1)	Invitrogen	#12-5381-82, RRID:AB_1907439	IF
RELN (polyclonal)	R&D systems	#AF3820, RRID:AB_2253745	IF
TCRb (H57-597)	BioLegend	#109208, RRID:AB_313430	FACS
TCRgd (GL3)	BioLegend	#118105, RRID:AB_313830	FACS
XCR1 (ZET)	BioLegend	#148213, RRID:AB_2564369	FACS
**Anti-human antibodies (clone)**			
CD3 (HIT3a)	BioLegend	#300323, RRID:AB_493738	FACS
CD45 (HI30)	BioLegend	#304031, RRID:AB_10900423	FACS
CD11c (Bu15)	BioLegend	#337235, RRID:AB_2566657	FACS
CD64 (10.a)	BioLegend	#305013, RRID:AB_1595428	FACS
EPCAM (9C4)	BioLegend	#324217, RRID:AB_10642820	FACS
TCRgd (B1.1)	eBioscience	#11-9959-41, RRID:AB_10669048	FACS
ARG1 (SP156)	Ventana Medical Systems	#760-4801	IHC
CD3 (2GV6)	Ventana Medical Systems	#790-4341	IHC
CD117	Dako	#A450229-2	IHC
CD207 (EP349)	Bio SB	#BSB 3375	IHC
CD34	Ventana Medical Systems	#760-2927	IHC
CK7 (SP52)	Ventana Medical Systems	#790-4462	IHC
CK19 (A53-B/A2.26)	Cell Marque	#760-4281	IHC
PDPN	Cell Marque	#760-4395	IHC
TRDC (E2E9T)	Cell Signalling	#55750	IHC
VIM (V9)	Ventana Medical Systems	#790-2917	IHC
**Anti-mouse TotalSeq Antibodies**			
F4/80 (BM8)	Biolegend (San Diego, USA), TotalSeq™-B0114	#123155, RRID:AB_2819847	proteogenomics

CD3 (17A2)	BioLegend, TotalSeq™-B0182	#100257, RRID:AB_2813912	proteogenomics
CD4 (RM4-5)	BioLegend, TotalSeq™-B0001	#100573, RRID:AB_2813914	proteogenomics
CD8a (53-6.7)	BioLegend, TotalSeq™-B0002	#100783, RRID:AB_2832269	proteogenomics
CD11b (M1/70)	BioLegend, TotalSeq™-B0014	#101273, RRID:AB_2819781	proteogenomics
CD80 (16-10A1)	BioLegend, TotalSeq™-B0849	#104757, RRID:AB_2888697	proteogenomics
CD115 (AFS98)	BioLegend, TotalSeq™-B0105	#135543, RRID:AB_2832487	proteogenomics
CD197 (4B12)	BioLegend, TotalSeq™-B0377	#120133, RRID:AB_2860644	proteogenomics
CD301b (URA-1)	BioLegend, TotalSeq™-B0566	#146821, RRID:AB_2888887	proteogenomics
CX3CR1 (SA011F11)	BioLegend, TotalSeq™-B0563	#149045, RRID:AB_2888877	proteogenomics
XCR1 (ZET)	BioLegend, TotalSeq™-B0568	#148231, RRID:AB_2888911	proteogenomics
Hashtag 3	BioLegend, TotalSeq™-B0303	#155835, RRID:AB_2814069	multiplexing
Hashtag 4	BioLegend, TotalSeq™-B0304	#155837, RRID: AB_2814070	multiplexing
Hashtag 5	BioLegend, TotalSeq™-B0305	#155839, RRID:AB_2814071	multiplexing
Hashtag 6	Biolegend, TotalSeq™-B0306	#155841, RRID:AB_2814072	multiplexing

Supplemental Table 6. Antibodies for FACS, IF, IHC and proteogenomics used in this study.

GEO Accession Numbers

Deposited Data	source	description
DDC atlas	this paper, GSE280985	DDC scRNA-seq data derived from male mice from liver (D0, D3, D5, D9, D19, D25, STOP, D5-DT)
human liver patient data	this paper, GSE280852	human pseudonormal liver scRNA-seq reference derived from 6 patients.
$\gamma\delta$T liver multiplex	this paper, GSE281197	$\gamma\delta$T enriched DDC dataset (D0, D5, D9, D19, STOP)
$\gamma\delta$T LN multiplex	this paper, GSE281197	$\gamma\delta$T enriched multiplexed DDC dataset comprising D0, D9, D18/19, STOP)
lymph node CITE seq	this paper, GSE281197	lymph node dataset of DDC D0, D5, D19 with co-detection of 11 proteins.
mouse cDC2 multiome	this paper, GSE281196	combined scRNA and scATAC sequencing data from ctrl (D0) and DDC D5 cDC2 (two experiments)
XENIUM human liver	https://www.10xgenomics.com/datasets/human-liver-data-xenium-human-multi-tissue-and-cancer-panel-1-standard	Human steady state liver
XENIUM DDC D5 liver	this paper, GSE311681	Xenium spatial transcriptomics data on DDC D5 using the Mouse Tissue Atlassing Panel in combination with a 100 gene add-on pool and the cell segmentation kit

Supplemental Table 7. GEO Accession numbers of deposited scRNA-seq and spatial transcriptomics data.

Software and Packages

Software and algorithms	Source/Reference	Version/Information
**BioRender**	https://app.biorender.com/	
**CellChat**	https://github.com/sqjin/CellChat [7]	v1
**CellRanger**	https://www.10xgenomics.com/support/software/cell-ranger/	v6.0.0
**FlowJo**	https://www.flowjo.com/	v10.7.1
**FIJI**	https://fiji.sc/ [8]	
**Kallisto**	https://pachterlab.github.io/kallisto/ [9]	
**NiCo**	https://github.com/ankitbioinfo/nico_tutorial [10]	v1.4
**NormqPCR**	https://bioconductor.org/packages/release/bioc/html/NormqPCR.html [11]	
**Python**	https://www.python.org/	3.9
**QuPath**	https://qupath.github.io/ [12]	QuPath-0.4.3
**R**	https://cran.r-project.org/index.html	4.4.0
**Seurat**	https://satijalab.org/seurat/ [13]	v5
**SCENIC**	https://scenic.aertslab.org/ [14]	
**Signac**	https://stuartlab.org/signac/articles/pbmc_vignette.html [15]	
**spacexr**	https://github.com/dmcable/spacexr [16]	
**VarID2**	https://cran.r-project.org/web/packages/RaceID/vignettes/RaceID.html [17]	

Supplemental Table 8. Software and packages.

Primers for qPCR (m)	Genbank ID	Sequence (5'-3')
**Actin (m)**	NM_007393	for: GCTTCTTTGCAGCTCCTTCGT
		rev: ACCAGCGCAGCGATATCG
**Acta2 (m)**	NM_007392	for: CTATTCAGGCTGTGCTGTCCCTC
		rev: CACGTTGTGAGTCACACCATCTC
**Adrgre1 (m)**	NM_010130	for: GCAGGTGCTGGAGAACATTCTAGAG
		rev: CCATCTCCTTCTTCATGTTGTCC
**Ccl17 (m)**	NM_011332	for: GTGAGCTGGTATAAGACCTCAGTG
		rev: CCTTCTTCACATGTTTGTCTTTGGG
**Ccl22 (m)**	NM_009137	for: CAAGAACCGAGATATCTGTGCCG
		rev: GTCCTCCTCCCTAGGACAGTTTATG
**Ccr2 (m)**	NM_009915	for: CCACCACACCGTATGACTACGATG
		rev: CAAGCTCCAATTTGCTTCACACTG
**Ccr7 (m)**	NM_007719	for: CTGGTGGTGGCTCTCCTTG
		rev: CGATGTAGTCATCGGTGACCTC
**Cd3e (m)**	NM_007648	for: CGTCTGCTACACACCAGCCTC
		rev: GTCCACCTCCACACAGTACTCAC
**Cd5l (m)**	NM_009690	for: CATGGCGAGGACACATGGATG
		rev: GGAACCCTTGTGTAGCACCTCC
**Cd11b (m)**	EF101557	for: CTCAACTTCACGGCTTCAGAGATG
		rev: GACTACACTGACAGGGAGGCTCCTC
**Cd24a (m)**	NM_009846	for: CACCGTTTCCCGGTAACCAG
		rev: CCTCTGGTGGTAGCGTTACTTGG
**Cd80 (m)**	NM_001359898	for: GCTCATTCTTCTCTTTGTGCTGCTG
		rev: GAGGAGAGTTGTAACGGCAAGG
**Cd209a (m)**	NM_133238	for: GGTAGATGGTTCACCTCTGACTCTC
		rev: CTTCCTCTCCCAGGTTGTTAGGTTC
**Clec4f (m)**	NM_016751	for: GCACAGACAACCAGTGTGTCTC
		rev: CAGAATGACCTGGACGTGCC
**Col1a1 (m)**	NM_007742	for: GTCTGGTTTGGAGAGAGCATGA
		rev: CGCAGGAAGGTCAGCTGGATAG
**Csf3r (m)**	NM_007782	for: GGAAGACAGCGTCCAACTCCTG
		rev: CTGTTGGTGGTGAGGTGCATG
**Cx3cr1 (m)**	NM_009987	for: GTCCACCTCCTTCCCTGAACTGG
		rev: GTCGCCCAAATAACAGGCCTCAG
**Des (m)**	NM_010043	for: CCATCGCGGCTAAGAACATCTCTG
		rev: CGCAGCGCATCGTTGTTCTTATTG
**Epcam (m)**	NM_008532	for: CCAGTGTACTTCCTATGGTACACAG
		rev: GCTTTCATCGCCAAGCATTTAGACG
**Gapdh (m)**	NM_008084	for: TGTCCGTCGTGGATCTGAC
		rev: CCTGCTTCACCACCTTCTTG
**Hprt (m)**	NM_013556	for: TCCTCCTCAGACCGCTTTT
		rev: CCTGGTTCATCATCGCTAATC
**Il17a (m)**	NM_010552	for: GTCCAGGGAGAGCTTCATCTG
		rev: GTCCAGGGAGAGCTTCATCTG
**Itgax (m)**	NM_021334	for: GAGCTGTACCTGGATAGCCTTTC
		rev: GTGTCAGCTTCTCTGCATCCAAG
**Krt7 (m)**	NM_033073	for: CAACAAATTCGCCTCCTTCATCGAC
		rev: CCCACTTGGTCTCCAGCATC
**Mgl2 (m)**	NM_145137	for: GTGGTCATCTCCGTGATTGGATC
		rev: GCCTAGGTCCCTCCTTAACTGG
**Pdgfrb (m)**	NM_001146268	for: GAGCTCAGTGAGAGGAAGCGTATC
		rev: GAACAGGTCCTCGGAGTCCATAG
**Ppia (m)**	NM_008907	for: GCATACAGGTCCTGGCATCT

		rev: AGCTGTCCACAGTCGGAAAT
**Sirpa (m)**	NM_001291019	for: GTTCCAGAAAGGATCATCAGAGCC
		rev: GATACCTCCGGTGGAGAAGGTTTG
**Siglech (m)**	NM_178706	for: GGAAAGGATCTCTGTGCATGTGAC
		rev: CGACCAAGCTCCAGTGTCTG
**Tlr7 (m)**	NM_001290755	for: GCCCATGTGATCGTGGACTG
		rev: GATGGTAAGGGTAAGATTGGTGGTG
**Tnf (m)**	NM_001278601	for: CGCTCTTCTGTCTACTGAACTTCGG
		rev: GTGTGAGGGTCTGGGCCATAG
**Trdc (m)**	NC_000080.7	for: GACACACAAATTTCAGAGCCTTGC
		rev: GTTTACCTTCTCAGTGTGAACTGTG
**Vsig4 (m)**	NM_177789	for: GCCAGGAAGACAAGCAACTCTG
		rev: GATGGTAGTCACCCTTGTGGTTTC
**Xcr1 (m)**	NM_011798	for: CAATGGAGTTGGTTTCTAGGTGAC
		rev: CCGAAGATCATGTTGAAGAACTTGC

Supplemental Table 9. Primer list for semi-quantitative real time PCR on mouse (m) samples.

References

1. Guilliams, M., C.A. Dutertre, C.L. Scott, et al., *Unsupervised High-Dimensional Analysis Aligns Dendritic Cells across Tissues and Species.* Immunity, 2016. **45**(3): p. 669-684. 10.1016/j.immuni.2016.08.015
2. Poch, T., J. Krause, C. Casar, et al., *Single-cell atlas of hepatic T cells reveals expansion of liver-resident naive-like CD4(+) T cells in primary sclerosing cholangitis.* J Hepatol, 2021. **75**(2): p. 414-423. 10.1016/j.jhep.2021.03.016
3. Kumamoto, Y., M. Linehan, J.S. Weinstein, B.J. Laidlaw, J.E. Craft, and A. Iwasaki, *CD301b(+) dermal dendritic cells drive T helper 2 cell-mediated immunity.* Immunity, 2013. **39**(4): p. 733-43. 10.1016/j.immuni.2013.08.029
4. Nakae, S., Y. Komiyama, A. Nambu, et al., *Antigen-specific T cell sensitization is impaired in IL-17-deficient mice, causing suppression of allergic cellular and humoral responses.* Immunity, 2002. **17**(3): p. 375-87. 10.1016/s1074-7613(02)00391-6
5. Itohara, S., P. Mombaerts, J. Lafaille, et al., *T cell receptor delta gene mutant mice: independent generation of alpha beta T cells and programmed rearrangements of gamma delta TCR genes.* Cell, 1993. **72**(3): p. 337-48. 10.1016/0092-8674(93)90112-4
6. Sandrock, I., A. Reinhardt, S. Ravens, et al., *Genetic models reveal origin, persistence and non-redundant functions of IL-17-producing gammadelta T cells.* J Exp Med, 2018. **215**(12): p. 3006-3018. 10.1084/jem.20181439
7. Jin, S., C.F. Guerrero-Juarez, L. Zhang, et al., *Inference and analysis of cell-cell communication using CellChat.* Nat Commun, 2021. **12**(1): p. 1088. 10.1038/s41467-021-21246-9
8. Schindelin, J., I. Arganda-Carreras, E. Frise, et al., *Fiji: an open-source platform for biological-image analysis.* Nat Methods, 2012. **9**(7): p. 676-82. 10.1038/nmeth.2019
9. Bray, N.L., H. Pimentel, P. Melsted, and L. Pachter, *Near-optimal probabilistic RNA-seq quantification.* Nat Biotechnol, 2016. **34**(5): p. 525-7. 10.1038/nbt.3519
10. Agrawal, A., S. Thomann, S. Basu, and D. Grun, *NiCo identifies extrinsic drivers of cell state modulation by niche covariation analysis.* Nat Commun, 2024. **15**(1): p. 10628. 10.1038/s41467-024-54973-w
11. Perkins, J.R., J.M. Dawes, S.B. McMahon, D.L. Bennett, C. Orengo, and M. Kohl, *ReadqPCR and NormqPCR: R packages for the reading, quality checking and normalisation of RT-qPCR quantification cycle (Cq) data.* BMC Genomics, 2012. **13**: p. 296. 10.1186/1471-2164-13-296
12. Bankhead, P., M.B. Loughrey, J.A. Fernandez, et al., *QuPath: Open source software for digital pathology image analysis.* Sci Rep, 2017. **7**(1): p. 16878. 10.1038/s41598-017-17204-5
13. Hao, Y., T. Stuart, M.H. Kowalski, et al., *Dictionary learning for integrative, multimodal and scalable single-cell analysis.* Nat Biotechnol, 2024. **42**(2): p. 293-304. 10.1038/s41587-023-01767-y
14. Aibar, S., C.B. Gonzalez-Blas, T. Moerman, et al., *SCENIC: single-cell regulatory network inference and clustering.* Nat Methods, 2017. **14**(11): p. 1083-1086. 10.1038/nmeth.4463
15. Stuart, T., A. Srivastava, S. Madad, C.A. Lareau, and R. Satija, *Single-cell chromatin state analysis with Signac.* Nat Methods, 2021. **18**(11): p. 1333-1341. 10.1038/s41592-021-01282-5
16. Cable, D.M., E. Murray, L.S. Zou, et al., *Robust decomposition of cell type mixtures in spatial transcriptomics.* Nat Biotechnol, 2022. **40**(4): p. 517-526. 10.1038/s41587-021-00830-w
17. Rosales-Alvarez, R.E., J. Rettkowski, J.S. Herman, G. Dumbovic, N. Cabezas-Wallscheid, and D. Grun, *VarID2 quantifies gene expression noise dynamics and unveils functional heterogeneity of ageing hematopoietic stem cells.* Genome Biol, 2023. **24**(1): p. 148. 10.1186/s13059-023-02974-1
