# Supplementary material for: An immunobiliary single-cell atlas resolves crosstalk between type 2 conventional dendritic cells and γδ T cells in cholangitis
Source: Nat Commun. 2026 Apr 10;17:3455. doi: 10.1038/s41467-026-71537-2 (PMC13076812; doi:10.1038/s41467-026-71537-2)
Supplement: Supplementary file 2 — Description of Additional Supplementary Files [file 41467_2026_71537_MOESM2_ESM.pdf]

Description of Additional Supplementary Files

Supplementary Data 1

Description: Supplementary data table 1 depicts the top 100 upregulated genes per cluster of the initial DDC scRNA-seq atlas (compare Fig. 1K). The VarID2 “clustdiffgenes” function was used to calculate upregulated genes using a p-value cutoff of 0.01. The table depicts gene names, mean expression in the cluster of interest (“mean.cl”) compared to all other clusters (“mean.ncl”). “pv” indicates p-value prior to multiple testing correction, while “padj” indicates the Benjamini-Hochberg corrected false discovery rate. Cluster IDs (“cluster”) as indicated in Fig. 1K.
