# Supplementary material for: An immunobiliary single-cell atlas resolves crosstalk between type 2 conventional dendritic cells and γδ T cells in cholangitis
Source: Nat Commun. 2026 Apr 10;17:3455. doi: 10.1038/s41467-026-71537-2 (PMC13076812; doi:10.1038/s41467-026-71537-2)
Supplement: Supplementary file 4 — Reporting Summary [file 41467_2026_71537_MOESM4_ESM.pdf]

nature portfolio

Corresponding author(s): Dominic Grün

Last updated by author(s): 17/2/26

Reporting Summary

Nature Portfolio wishes to improve the reproducibility of the work that we publish. This form provides structure for consistency and transparency in reporting. For further information on Nature Portfolio policies, see our Editorial Policies and the Editorial Policy Checklist.

Statistics

For all statistical analyses, confirm that the following items are present in the figure legend, table legend, main text, or Methods section.

n/a	Confirmed	
☐	☒	The exact sample size (*n*) for each experimental group/condition, given as a discrete number and unit of measurement
☐	☒	A statement on whether measurements were taken from distinct samples or whether the same sample was measured repeatedly
☐	☒	The statistical test(s) used AND whether they are one- or two-sided<br>*Only common tests should be described solely by name; describe more complex techniques in the Methods section.*
☒	☐	A description of all covariates tested
☐	☒	A description of any assumptions or corrections, such as tests of normality and adjustment for multiple comparisons
☐	☒	A full description of the statistical parameters including central tendency (e.g. means) or other basic estimates (e.g. regression coefficient) AND variation (e.g. standard deviation) or associated estimates of uncertainty (e.g. confidence intervals)
☐	☒	For null hypothesis testing, the test statistic (e.g. *F*, *t*, *r*) with confidence intervals, effect sizes, degrees of freedom and *P* value noted<br>*Give P values as exact values whenever suitable.*
☒	☐	For Bayesian analysis, information on the choice of priors and Markov chain Monte Carlo settings
☒	☐	For hierarchical and complex designs, identification of the appropriate level for tests and full reporting of outcomes
☒	☐	Estimates of effect sizes (e.g. Cohen's *d*, Pearson's *r*), indicating how they were calculated

Our web collection on statistics for biologists contains articles on many of the points above.

Software and code

Policy information about availability of computer code

Data collection	Sequencing data was mapped using kallisto or CellRanger (v6.0.0). All used software tool and further information regarding version number etc. can be found in Supplemental Table 8. Human spatial transcriptomics data were downloaded from 10X Genomics Datasets (link provided in Supplemental Table 7.
Data analysis	scRNA-seq data analysis was conducted with VarID2, Seurat (v3/v5), NiCo (v1.4), CellChat (v1), Signac, SCENIC, spacexr using R (v4.4.0) or Python (v3.9).. A full list of all packages being used in this manuscript is part of the supplemental information. FlowJo (v10) was used for FACS data. FIJI (v1.53j) and QuPath (v0.4.3) were used for microscopy images/scans.

For manuscripts utilizing custom algorithms or software that are central to the research but not yet described in published literature, software must be made available to editors and reviewers. We strongly encourage code deposition in a community repository (e.g. GitHub). See the Nature Portfolio guidelines for submitting code & software for further information.

Data

Policy information about availability of data

All manuscripts must include a data availability statement. This statement should provide the following information, where applicable:

- Accession codes, unique identifiers, or web links for publicly available datasets
- A description of any restrictions on data availability
- For clinical datasets or third party data, please ensure that the statement adheres to our policy

All generated single-cell sequencing data are available at GEO as raw and processed data under the following accession numbers: human liver data, GSE280852; mouse DDC atlas, GSE280985; Multiome data, GSE281196; CITE-seq/Hash-multiplexed data, GSE281197. DDC D5 spatial transcriptomics data is accessible at GSE 311681. Source Data are provided with this paper. The human tissue microarray data cannot be made accessible due to institute-specific guidelines and human ethics regulations.

scRNA-seq data was uploaded on GEO and are publicly accessible as of February 17, 2026. Human Xenium Liver data and its accessibility are listed in the manuscript (Table S7).
GEO accession numbers are listed below (data publicly released):

Human Liver Data https://www.ncbi.nlm.nih.gov/geo/query/acc.cgi?acc=GSE280852

Initial DDC Atlas https://www.ncbi.nlm.nih.gov/geo/query/acc.cgi?acc=GSE280985

Multiome Data https://www.ncbi.nlm.nih.gov/geo/query/acc.cgi?acc=GSE281196

CITE-seq and Hash-multiplexed Data https://www.ncbi.nlm.nih.gov/geo/query/acc.cgi?acc=GSE281197

DDC D5 spatial transcriptomics data is accessible at GSE 311681

Research involving human participants, their data, or biological material

Policy information about studies with human participants or human data. See also policy information about sex, gender (identity/presentation), and sexual orientation and race, ethnicity and racism.

Reporting on sex and gender	Male mice were used unless otherwise stated.
Reporting on race, ethnicity, or other socially relevant groupings	Not considered in the study design.
Population characteristics	Clinical information such as age and gender of human scRNA-seq data are listed in the methods section (Fig. S1A).
Recruitment	Not considered in the study design.
Ethics oversight	Ethics vote is included for the retrospective tissue analysis (waiver for patient consent).

Note that full information on the approval of the study protocol must also be provided in the manuscript.

Field-specific reporting

Please select the one below that is the best fit for your research. If you are not sure, read the appropriate sections before making your selection.

[x] Life sciences [ ] Behavioural & social sciences [ ] Ecological, evolutionary & environmental sciences

For a reference copy of the document with all sections, see nature.com/documents/nr-reporting-summary-flat.pdf

Life sciences study design

All studies must disclose on these points even when the disclosure is negative.

Sample size	Number of single cell datasets and number of cells provided in the respective section of the manuscript.
Data exclusions	No data was excluded.
Replication	Statistical parameters were provided in the figures.
Randomization	Not applicable.
Blinding	Not applicable.

Reporting for specific materials, systems and methods

We require information from authors about some types of materials, experimental systems and methods used in many studies. Here, indicate whether each material, system or method listed is relevant to your study. If you are not sure if a list item applies to your research, read the appropriate section before selecting a response.

Materials & experimental systems

n/a	Involved in the study
[ ]	[x] Antibodies
[x]	[ ] Eukaryotic cell lines
[x]	[ ] Palaeontology and archaeology
[ ]	[x] Animals and other organisms
[ ]	[x] Clinical data
[x]	[ ] Dual use research of concern
[x]	[ ] Plants

Methods

n/a	Involved in the study
[x]	[ ] ChIP-seq
[ ]	[x] Flow cytometry
[x]	[ ] MRI-based neuroimaging

Antibodies

Antibodies used

All antibodies used for FACS, IF and for CITE-seq are listed in the Supplemental Methods (Table S6). Antibodies were tested and concentrations used according to the manufacturer's recommendations.

Anti-mouse antibodies (clone)
CD3e (145-2C11) BD Biosciences #553061, RRID:AB_394594 FACS/IF
CD11b (M1/70) Affymetrix eBioscience #11-0112-82, RRID:AB_464935 FACS/IF
CD11c (N418) BioLegend #117309, RRID:AB_313778 FACS/IF
CD16/32 Biolegend #101330, RRID:AB_2561482 FACS
CD19(MB19-1) BioLegend #101506, RRID:AB_312825 FACS
CD31 (MEC13.3) BioLegend #102429, RRID:AB_2566206 FACS/IF
CD44 (IM7) BioLegend #103047, RRID:AB_2562451 FACS
CD45 (30-F11) BioLegend #103108, RRID:AB_312972 FACS/IF
CD62L (MEL-14) BioLegend #104447, RRID:AB_2566162 FACS
CD64 (X54-5/7.1) BioLegend #139323, RRID:AB_2629778 FACS
CD146 (ME-9F1) BD Biosciences #740095, RRID:AB_2739854 FACS/IF
CD172a/SIRPα (P84) BioLegend #144023, RRID:AB_2650815 FACS/IF
CD278 (ICOS) eBioscience # 14-9949-82, RRID:AB_468637 functional
CD301b (URA-1) Biolegend #146803, RRID:AB_2562943 FACS/IF
CD335 (NKp46) BioLegend #137605, RRID:AB_2149150 FACS
EPCAM (G8.8) eBioscience #17-5791-82, RRID:AB_2716944 FACS/IF
F4/80 (REA126) MiltenyiBiotec #130-117-509, RRID:AB_2727970 FACS/IF
Il17a (TC11-18H10.1) BioLegend #506933, RRID:AB_2562668 FACS
LYVE-1 (ALY7) eBioscience #53-0443-82, RRID:AB_1633415 FACS/IF
MGL1/2 R&D systems #AF4297, RRID:AB_2248147 IHC
MHC class 2 (M5/114.15.2) eBioscience #78-5321-82, RRID:AB_2744917 FACS
PDGFRB Cell Signaling #3169, RRID:AB_2162497 IHC
PDPN (eBio8.1.1) Invitrogen #12-5381-82, RRID:AB_1907439 IF
RELN (polyclonal) R&D systems #AF3820, RRID:AB_2253745 IF
TCRb (H57-597) BioLegend #109208, RRID:AB_313430 FACS
TCRgd (GL3) BioLegend #118105, RRID:AB_313830 FACS
XCR1 (ZET) BioLegend #148213, RRID:AB_2564369 FACS

Anti-human antibodies (clone)
CD3 (HIT3a) BioLegend #300323, RRID:AB_493738 FACS
CD45 (HI30) BioLegend #304031, RRID:AB_10900423 FACS
CD11c (Bu15) BioLegend #337235, RRID:AB_2566657 FACS
CD64 (10.a) BioLegend #305013, RRID:AB_1595428 FACS
EPCAM (9C4) BioLegend #324217, RRID:AB_10642820 FACS
TCRgd (B1.1) eBioscience #11-9959-41, RRID:AB_10669048 FACS
ARG1 (SP156) Ventana Medical Systems #760-4801 IHC
CD3 (2GV6) Ventana Medical Systems #790-4341 IHC
CD117 Dako #A450229-2 IHC
CD207 (EP349) Bio SB #BSB 3375 IHC
CD34 Ventana Medical Systems #760-2927 IHC
CK7 (SP52) Ventana Medical Systems #790-4462 IHC
CK19 (A53-B/A2.26) Cell Marque #760-4281 IHC
PDPN Cell Marque #760-4395 IHC
TRDC (E2E9T) Cell Signalling #55750 IHC
VIM (V9) Ventana Medical Systems #790-2917 IHC
Anti-mouse TotalSeq Antibodies
F4/80 (BM8) Biolegend (San Diego, USA), TotalSeq™-B0114 #123155, RRID:AB_2819847 proteogenomics
CD3 (17A2) BioLegend, TotalSeq™-B0182 #100257, RRID:AB_2813912 proteogenomics

	CD4 (RM4-5) BioLegend, TotalSeq™-B0001 #100573, RRID:AB_2813914 proteogenomics<br>CD8a (53-6.7) BioLegend, TotalSeq™-B0002 #100783, RRID:AB_2832269 proteogenomics<br>CD11b (M1/70) BioLegend, TotalSeq™-B0014 #101273, RRID:AB_2819781 proteogenomics<br>CD80 (16-10A1) BioLegend, TotalSeq™-B0849 #104757, RRID:AB_2888697 proteogenomics<br>CD115 (AFS98) BioLegend, TotalSeq™-B0105 #135543, RRID:AB_2832487 proteogenomics<br>CD197 (4B12) BioLegend, TotalSeq™-B0377 #120133, RRID:AB_2860644 proteogenomics<br>CD301b (URA-1) BioLegend, TotalSeq™-B0566 #146821, RRID:AB_2888887 proteogenomics<br>CX3CR1 (SA011F11) BioLegend, TotalSeq™-B0563 #149045, RRID:AB_2888877 proteogenomics<br>XCR1 (ZET) BioLegend, TotalSeq™-B0568 #148231, RRID:AB_2888911 proteogenomics<br>Hashtag 3 BioLegend, TotalSeq™-B0303 #155835, RRID:AB_2814069 multiplexing<br>Hashtag 4 BioLegend, TotalSeq™-B0304 #155837, RRID: AB_2814070 multiplexing<br>Hashtag 5 BioLegend, TotalSeq™-B0305 #155839, RRID:AB_2814071 multiplexing<br>Hashtag 6 Biolegend, TotalSeq™-B0306 #155841, RRID:AB_2814072 multiplexing
Validation	All antibodies are commercially available and have been validated by the manufacturers.

Animals and other research organisms

Policy information about studies involving animals; ARRIVE guidelines recommended for reporting animal research, and Sex and Gender in Research

Laboratory animals	Mouse strains indicated were used and male mice used within the range of the age of 6 - 12 weeks. Mice were bred in a 12-hour light/dark cycle with free access to water and food and kept under defined pathogen free conditions with littermates receiving control chow being kept and housed in separate cages in the same facility room. Ambient temperature ranged between 20-24°C and the humidity range was 30-70%.<br>Strains:<br>C57BL/6J C57BL/6JRj Janvier, RRID:IMSR_RJ:C57BL-6JRJ<br>CD301b-DTR (Mgl2-DTR), B6(FVB)Mgl2tm1.1(HBEGF/EGFP)Aiwsk/J #023822 (Jackson), RRID:IMSR_JAX:023822<br>Il17a_f_KO, B6.129P2-Il17atm1Yiw Il17ftm2Yiw #RRID: MGI:3830065<br>Tcrdtm_KO, B6.129P2-Tcrd tm1 Mom/J #002120 (Jackson), RRID:IMSR_JAX:002120<br>Tcrd- GDL, C57BL/6N-Trdctm1(EGFP/HBEGF/luc)Impr/J Strain #:038040, RRID:IMSR_JAX:038040
Wild animals	Study did not involve wild animals.
Reporting on sex	Male mice were used when not indicated otherwise. Only for cDC2 FACS quantifications female mice were used. Genetic background, age, gender are stated in the main manuscript.
Field-collected samples	Not applicable.
Ethics oversight	All animal experiments were approved and conducted under the regulations of the local Government (Regierung Unterfranken, licence RUF 2-1554 and Regierungspräsidium Freiburg, licence G20/164).

Note that full information on the approval of the study protocol must also be provided in the manuscript.

Clinical data

Policy information about clinical studies
All manuscripts should comply with the ICMJE guidelines for publication of clinical research and a completed CONSORT checklist must be included with all submissions.

Clinical trial registration	Ethics vote numbers covering all analyses conducted in the manuscript: S-230/20, S-206, 207/05, 2012-293N-MA and 2021-2320-1. Ethics vote were approved from the ethics committee in Heidelberg and Jena.
Study protocol	Not relevant.
Data collection	All votes included the right to use either FFPE embedded tissue of patients were the diagnostics procedure is finished, so that material can be used without any adverse consequences of the patient. Fresh liver tissues were provided from resection specimens where tissue had to be removed based on a medical indication, however not all tissue will be used for follow up diagnostics.
Outcomes	The votes do not cover any prospective study elements, which is why an outcome is not applicable.

Plants

Seed stocks	Not applicable
Novel plant genotypes	Not applicable
Authentication	Not applicable

Flow Cytometry

Plots

Confirm that:

☒ The axis labels state the marker and fluorochrome used (e.g. CD4-FITC).

☒ The axis scales are clearly visible. Include numbers along axes only for bottom left plot of group (a 'group' is an analysis of identical markers).

☒ All plots are contour plots with outliers or pseudocolor plots.

☒ A numerical value for number of cells or percentage (with statistics) is provided.

Methodology

Sample preparation	Sample preparation for sorting or for FACS analysis is described in the Methods section.
Instrument	Instruments are listed in the Methods section.
Software	FlowJo Software (v10) was used.
Cell population abundance	Cell Population abundance is indicated in all FACS plots and FACS sorting strategy for scRNA-seq data provides information about the proportion of cells being used.
Gating strategy	Gating strategy is provided.

☒ Tick this box to confirm that a figure exemplifying the gating strategy is provided in the Supplementary Information.
